# BMI and BMD: The Potential Interplay between Obesity and Bone Fragility

**DOI:** 10.3390/ijerph13060544

**Published:** 2016-05-28

**Authors:** Andrea Palermo, Dario Tuccinardi, Giuseppe Defeudis, Mikiko Watanabe, Luca D’Onofrio, Angelo Lauria Pantano, Nicola Napoli, Paolo Pozzilli, Silvia Manfrini

**Affiliations:** 1 Department of Endocrinology and Diabetes, University Campus Bio-Medico, Via Alvaro del Portillo, 21, Rome 00128, Italy; d.tuccinardi@unicampus.it (D.T.); g.defeudis@unicampus.it (G.D.); a.pantano@unicampus.it (A.L.P.); n.napoli@unicampus.it (N.N.); p.pozzilli@unicampus.it (P.P.); s.manfrini@unicampus.it (S.M.); 2 Department of Experimental Medicine, Sapienza University of Rome, Rome 00185, Italy; mikiko.watanabe@gmail.com; 3 Department of Experimental Medicine, Polo Pontino, Sapienza University of Rome, Rome 00185, Italy; luca.donofrio@live.com; 4 Centre of Diabetes, Barts & the London School of Medicine, London E1 2AT, UK

**Keywords:** osteoporosis, obesity, inflammation, fracture, body composition

## Abstract

Recent evidence demonstrating an increased fracture risk among obese individuals suggests that adipose tissue may negatively impact bone health, challenging the traditional paradigm of fat mass playing a protective role towards bone health. White adipose tissue, far from being a mere energy depot, is a dynamic tissue actively implicated in metabolic reactions, and in fact secretes several hormones called adipokines and inflammatory factors that may in turn promote bone resorption. More specifically, Visceral Adipose Tissue (VAT) may potentially prove detrimental. It is widely acknowledged that obesity is positively associated to many chronic disorders such as metabolic syndrome, dyslipidemia and type 2 diabetes, conditions that could themselves affect bone health. Although aging is largely known to decrease bone strength, little is yet known on the mechanisms via which obesity and its comorbidities may contribute to such damage. Given the exponentially growing obesity rate in recent years and the increased life expectancy of western countries it appears of utmost importance to timely focus on this topic.

## 1. Introduction

Obesity and osteoporosis are two of the most important diseases strictly related with an increased prevalence in both mortality and morbidity worldwide [1,2,3,4,5]. Different studies have shown a protective role of obesity against osteoporosis but recent evidence suggests that obesity, and thus fat mass, may prove to be risk factors for decreased bone density and fractures [6,7,8].

Obesity can be defined as a complex disorder involving an abnormal or excessive amount of body fat. This imbalance increases the risk associated with different diseases such as heart disease, diabetes and high blood pressure.

The World Health Organization (WHO) underlined that in 2014 more than 1.9 billion adults were overweight and, of these, over 600 million were obese. About 13% of the world’s adult population (11% of men and 15% of women) were obese in 2014. Moreover, the worldwide prevalence of obesity has more than doubled between 1980 and 2014. There are many potential causes for this condition of energy imbalance between calories consumed and calories burned. Among these potential causes, we can suggest an increased intake of energy-dense foods that are high in fat, and a decrease in physical activity due to a change in lifestyle habits such as sedentary work, increased use of automated means of transportation, and increasing urbanization [9].

In the United States (U.S.) it is estimated that obesity costs range from $147 billion to nearly $210 billion per year [10,11]. Job absenteeism, costing approximately $4.3 billion annually [12] and lower productivity while at work, have a cost to employers of approximately $506 per obese worker per year [13]. What about Europe? Obesity-related healthcare burdens of up to €10.4 billion were found and relative economic burdens ranged from 0.09% to 0.61% of each country’s gross domestic product [14].

Osteoporosis can be defined as a skeletal disorder characterized by compromised bone strength which leads to an increased risk of fracture. Bone strength reflects the integration of two main features: bone density and bone quality. Osteoporosis is the most common underlying cause of fractures and accounts for approximately 1.5 million fractures in the U.S. each year [15,16]. This bone condition is defined on the basis of Bone Mineral Density (BMD) assessment. According to WHO criteria, osteoporosis is defined as a condition in which BMD lies 2.5 standard deviations or more below the average value for young healthy women (a T-score of ≤2.5 SD) [17]. In the future, given the rise in median age, we expect a significant increase in the prevalence of osteoporosis and associated rate of fractures [16]. By 2020, over 14 million subjects older than 50 could be affected by osteoporosis and another 47 million could have low bone mass [17]. The country of origin or ethnicity plays a crucial role, and European Americans have the greatest reported risk [18,19,20]. Furthermore, over 500,000 hospitalizations, more than 2.6 million medical visits, over 800,000 emergency room admittances and approximately 180,000 individuals being placed into nursing homes are registered every year in the U.S. [21], anticipating an increase in costs by 100% to 200% by 2040 [21].

Historically, obesity has been linked to bone health as a protective factor [22,23]. Nonetheless, adipose tissue represents less than 40% of total body weight on average, meaning that the mechanical load related to increased fat mass may be insufficient to induce this positive effect on bone tissue [24]. Hence, recent studies have been conducted to re-evaluate whether obese individuals may or may not have an increased risk of presenting certain types of fracture by anatomical zone [25]. With worldwide increases in both Body Mass Index (BMI) and age, it has never been more important to understand the risks of osteoporosis in this population [26].

The aim of this review is to analyse and clarify the interplay between BMI, BMD and risk of fractures.

## 2. Materials and Methods

**Figure 1:**
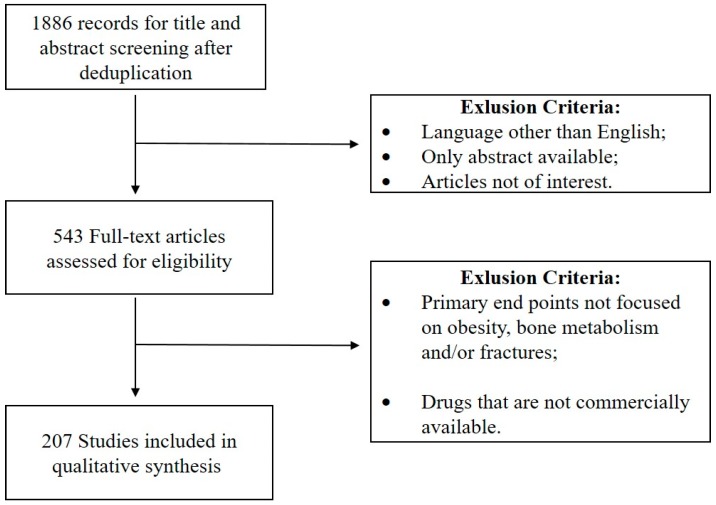
The process of study selection.

PubMed and MEDLINE were searched conforming to PRISMA guidelines [27] in order to identify publications about obesity and bone health. The study selection process is illustrated in Figure 1. Specifically, we considered those that examined the potential relationship between obesity and bone impairment and questioned how obesity may affect bone metabolism. Obesity, BMI and adipokines were matched with BMD, bone quality, bone biomarkers and bone fractures. Only publications in English only were included. 

## 3. Interplay between BMI and BMD: Epidemiology of Fracture Risk in Obese Subjects

As mentioned above, obesity is traditionally linked to increased bone strength and lower fracture risk. Many large studies have corroborated this assumption in several populations. However, recent evidence suggests that this should not be taken as a dogma. In fact, many different factors account for the interplay between BMI and BMD, thus giving space for ample discussion.

In order to better understand the crosstalk between bone and adipose tissue, it is crucial to adequately interpret the results presented in literature. Most of the existing data confirm that adipose tissue has an independent effect on bone remodeling leading to an increase in bone mass. Mechanisms accounting for this relationship may be, for example, mechanical load that in turn stimulates bone formation [28], androgens-to-estrogens conversion in adipose tissue, lower serum levels of Sex Hormone Binding Globulin (SHBG) [29], increased serum leptin levels [30], increased insulin growth factor production and hyperinsulinemia [31].

While a BMI <18.5 kg/m^2^ in older people has been widely linked to an increased risk of fracture [32,33,34,35,36], it has yet to be clarified whether the relationship between adiposity and risk of fracture is correlated with BMI. Data from the Study of Osteoporotic Fractures showed that total body weight, fat mass, body fat percentage, hip girth and BMI were inversely associated with fracture risk before correction for BMD. When adjustment for BMD was performed, the relationship appeared to be U-shaped [37], confirming that the effect of BMI on fracture risk is nonlinear. According to this data, we may infer that an increase in BMI above eutrophic ranges is weakly protective against fractures but this effect tends to disappear as we move towards morbid obesity. However, more evidence is needed to reach a definitive conclusion.

Manzoni *et al.* have reported that obese children and adolescents had higher Total and Regional Bone Mineral Content (TBMC and RBMC, respectively) when compared to lean children. However, those differences were no longer significant after correction for potentially confounding variables such as age and gender [38]. A small study conducted by Fisher and colleagues showed that obese children had higher TBMC than eutrophic children, but no significant differences were found in hip or lumbar spine BMD between those two groups [39]. Correa Rodriguez *et al.* recently evaluated 157 adolescents by calcaneal osteosonography reporting that overweight and obese subjects had higher levels of broadband ultrasound attenuation even after correcting for lean and fat mass [40]. A very recent Iranian study confirmed this data with 472 adolescents whose BMD was evaluated by Dual X Ray Absorptiometry (DXA) scan. Obese individuals were found in fact to have greater total body BMD than normal-weight ones [41].

While there has been some evidence showing that increased weight increases bone health in children and adolescents, there have been many studies reporting contrasting data. For instance, Goulding *et al.* demonstrated that overweight and obese children do not increase their spinal BMC to fully compensate for their excess weight. This study was conducted on 362 children and adolescents evaluated by DXA scan [42]. The same authors later showed that obese children also had higher BMC, bone area, and fat mass for chronological age when compared to lean age-matched subjects but the observed values for age-adjusted total body BMC and bone area relative to body weight were lower than predicted values [43], underlining the need for careful corrections for fat mass. Moreover, Wetzsteon at al. highlighted that overweight children had greater bone strength than lean children when evaluated by pQCT but this was disproportionate to body mass [44]. A large, U.S.-based, cross-sectional study showed that increased BMI was associated with increased fracture risk for the foot, ankle, leg, and knee in children and adolescents [45]. A recent meta-analysis compiled by Paulis *et al.* confirmed that among adolescents increased BMI was associated with higher odds for injuries and fractures, although the evidence was reported not to be of high quality [46]. Another study, conducted by Taylor at al., explained that another reason why obesity may increase fracture risk is that obese children and adolescents have poorer mobility and balance [47]. Moreover, Davidson *et al.* reported that obese adolescents falling on outstretched limbs impose greater force to bones and are thus at greater risk for fractures [48].

The lack of a standardized way to assess bone mass and quality in this category of patients limits the conclusions that may be drawn, but, taking into account the evidence shown above, childhood obesity appears a condition where bone strength is in fact slightly increased but not enough to be able to cope with the resultant higher mechanical load and poorer mobility, resulting in an overall increased fracture risk.

Regarding the relationship between excess weight and bone health in premenopausal women, there is a lack of recent data. Cohen and colleagues reported that trunk fat, evaluated by DXA scan, was inversely associated with trabecular bone volume and bone formation rate, observed with a transiliac bone biopsy, even after controlling for age and BMI [49]. Bredella *et al.* indirectly confirm such a finding, reporting an inverse association between VAT and L4 trabecular BMD [50]. Ishii and colleagues showed a linear association between BMI and BMD but an inverse one between BMI and composite strength indexes, suggesting that even though BMD increases with weight, this is not able to compensate for increased impact forces during falls [51].

Further studies are needed to prove the link between bone and fat in premenopausal women, but what is shown above may partially confirm what is seen in other groups of patients, where visceral and subcutaneous fat seem to play different roles and BMI appears to be able to increase bone mass in some insufficient measure.

Evidence in men appears to be less diverse than it is for other populations. Still, there is some controversy. A population-based cohort study from Spain conducted on over 100,000 men aged 65 years and older showed a statistically significant reduction in clinical spine and hip fractures in obese and overweight individuals compared with lean ones. Also, obese men had significantly fewer wrist and forearm fractures than nonobese ones. Conversely, the risk of incurring multiple rib fractures was directly proportional to BMI [52]. Nielson *et al.* studied a cohort of 5995 U.S. men aged 65 years and older concluding that the risk of incurring vertebral fractures was directly proportional to BMI when adjusting for potential confounders such as age, race and BMD. However, these associations were dependent on mobility limitations and walking pace and they appeared non statistically significant when controlled for these confounders [26]. Shen and colleagues conducted a cross-sectional study on 3067 men from the Osteoporotic Fractures in Men Study (MrOS) analysing the relationship between BMI and hip QCT measures. Finite element analysis of hip QCT scans was performed for a subgroup of 672 men providing a measure of hip strength in a simulated fall. Although obese men showed a higher hip strength they also had a higher ratio of impact force to strength, theoretically increasing their risk of hip fracture despite the stronger bones [53]. A prospective cohort study from Norway of 23,061 men aged 60 to 79 years showed that fracture risk was lower with increasing BMI, coming to a plateau in obese men. However, higher waist circumference and higher waist-to-hip ratio were associated with an increased hip fracture risk when adjusted for BMI and other potential confounders. In fact, men in the highest tertile of waist circumference had a 100% (95% CI 51%–129%) higher risk of hip fracture compared with the lowest. Combining lower BMI with abdominal adiposity increased the risk of hip fracture considerably [54].

Again, it is not yet possible to draw a definitive conclusion regarding the bone-fat link in men, but the available literature seems to echo what has been observed in other categories, especially regarding the roles of differently localized adipose depots.

The majority of studies on this topic have been conducted on postmenopausal women and the elderly. Paganini-Hill and colleagues reported that high BMI was associated with a significant reduction in hip fracture risk independently of other potential confounders using data from 8600 postmenopausal women [55]. These data were confirmed by Cummings *et al.* in 9516 white women 65 years of age and older [56] and by Di Pietro and colleagues in 2285 women aged 55 to 77 years, where subjects with a BMI in the highest quartile (>37 kg/m^2^) had a 70% lower rate of hip fractures when compared with those in the lowest quartile (≤28.7 kg/m^2^) [57]. Accordingly, The Tromsø Study, a cohort study which included 12,097 subjects (almost half of it made of postmenopausal women), reported that overweight and obesity in women were significantly associated with a lower risk of all fractures [58]. A Dutch study on 4725 postmenopausal women also showed that patients who had been obese at their younger ages seemed to have a much lower lifetime fracture risk [59]. The European Vertebral Osteoporosis Study (EVOS), in which 16,047 subjects aged 50 years and older (50% women) were evaluated, confirmed previous observations by showing a trend of decreasing prevalence of vertebral deformity with increasing BMI in the female subgroup. Also the risk of incurring distal forearm fractures for those whose BMI was >25 kg/m^2^ appeared to be decreased by 36% in a sample of 11,798 women, 68% of whom postmenopausal [60]. A metanalysis published in 2005 [37] evaluated twelve prospective population-based cohorts (14,887 men and 44,757 women, with a mean age of 62.2 years). Low BMI in both men and women correlated with an increased age-adjusted risk of any type of fracture, whereas higher BMI values decreased the risk of fracture. However, the risk increase was not linear as the gradient risk per unit BMI was relatively low in the eutrophic range. The gradient appeared instead much steeper at lower BMI values. When the risk for fracture was adjusted for BMD, BMI appeared not to be a predictor except for hip fracture in the underweight range. In contrast, obesity was associated with a 17% reduction in hip fracture risk when compared with those of normal weight subjects, showing a more modest reduction in the risk of fracture compared to the risk decrease between underweight and normal weight conditions.

In contrast with what has been reported above, Watts *et al.* recently published data on 60,393 postmenopausal women showing that higher BMIs related to an increased risk of ankle and upper leg fractures, whereas wrist fractures were more common in lean subjects [61]. A Spanish study, which included 832,775 postmenopausal women demonstrated that there was a higher risk of proximal humerus fractures in obese compared to normal and underweight women. Nonetheless, the same study showed that the lean group had more hip and pelvis fractures. These associations did not change after adjustment for several confounders [62]. Confirming these findings, a recent meta-analysis pooling data from twenty five prospective cohorts for a total of 398,610 women evaluated aged 20–105 years with a mean age of 63 years showed that increased BMI was positively associated with a higher risk of upper arm fractures when this correlation was adjusted for BMD. Also, obesity was an independent risk factor for all osteoporotic fractures [63].

We have shown that a large majority of the evidence regarding the interplay between bone and adipose tissue is composed of large observational studies that, by nature, cannot assess causality and have intrinsic limitations. The link between bone and fat is complex and not yet thoroughly understood, but what we can infer from the literature available up-to-date is that not all fats are the same and not all fractures are alike. Obesity has proven to be both protective and detrimental to bone health and so its comorbidities must be taken into account to explain the whole picture. Studies are summarized in Table 1 and Table 2.

## 4. Physiopathology of the Bone-Body Cross Talk

Recent evidence on increased fracture risk in obese patients has fueled new interest in better understanding the mechanisms of bone physiopathology, particularly regarding the relationship between adipose and bone tissue.

Osteoblasts and adipocytes derive from a common mesenchymal stem cell. While osteoblastogenesis is induced by the Wnt/β-catenin signaling pathway, peroxisome proliferator-activated receptor gamma (PPAR-γ) is responsible for the differentiation of adipose tissue. In fact, bone marrow-derived mesenchymal stem cells treated *in vitro* with PPAR-γ and interleukin-1 (which suppresses its function) showed an inhibition of the adipogenesis pathway and a switch to the osteoblastogenesis one, confirming PPAR-γ as an essential component of adipose tissue differentiation [64].

PPAR-γ activity could thus be involved in the age-related bone marrow fat accumulation associated with suppressed production of osteoblasts and decreases in bone mass [65].

Moreover, PPAR-γ mRNA expression in adipose tissue is increased in obese subjects, suggesting that its more intense activity may be involved in reduced bone formation [66,67]. The activity of PPAR-γ also appears to be implicated in body fat distribution according to evidence from animal studies [68]. In fact, not all fat depots are the same: location [69,70] and type [71] of excessive adipose tissue, rather than simply total body adiposity, may be crucial in the systemic increase of circulating cytokines and the upsurge of metabolic diseases such as diabetes [71,72].

Subcutaneously stored adipose tissue depots, particularly those in the gluteal-femoral region, are negative predictors of metabolic syndrome and appear to be cardioprotective [73,74]. However, those stored in ectopic locations such as muscle, liver and abdominal cavity are linked with chronic inflammation [75,76], impaired glucose tolerance [77,78], increased total cholesterol [75,76,79] and decreased strength and mobility in older adults [80,81]. Advancing age results in a redistribution of fat depots, despite stable or decreasing overall fat, with adipose storage sites switching from subcutaneous locations to more harmful ectopic ones [69,82,83]. This process is also known as “the overflow hypothesis” [84]. Moreover, fat tissue location and distribution relate to several bone health parameters in healthy premenopausal women independently of obesity *per* *se* [50,85]. Recent evidence suggests that abdominal fat, VAT and bone marrow adipose tissue are associated with lower BMD, greater cortical porosity, lower bone formation rate and lower bone trabecular volume and stiffness. In contrast, subcutaneous adipose tissue (SAT) appears to be protective or neutral regarding bone health.

A shift in allocation of resources from bone to other compartments and vice versa is mediated by a cross communication between all fat compartments, several organs and bone tissue. The endocrine system, inflammation, and adipokines may be some of the components of such coordination.

It is known that during perimenopause a gradual decrease in estrogen levels occurs. The link between estrogen deficiency and accelerated bone loss has been well documented. Obese women show lower serum levels of SHBG thus leading to higher levels of free hormones compared with normal-weight women [86]. Higher adrenal production of androstenedione with a subsequent increased pool of precursors ready for peripheral conversion is observed in these subjects as well [87]. As aromatase expression also increases with age in adipocytes [88], fat tissue activity in terms of estrogens production is one of the potential mechanisms that can explain the protective effect of obesity on bone health.

Although the relationship between estrogen metabolism and bone tissue is well established, less is known about estrogens and body composition. Napoli *et al.* [89] showed that an increase in the metabolism of estrogen towards the inactive metabolites is associated with lower body fat and higher lean mass. These results suggest that a subset of women with a specific pattern of estrogen metabolism may be somewhat protected from obesity, leading to both advantages and disadvantages of this condition.

Research in the last decade has revealed that bone tissue has connections with several other circulating hormones [90]. Osteocalcin (Ocn), an osteoblast-derived hormone considered a marker of bone formation but also released from the bone matrix during the resorption phase [91], stimulates testosterone production in mice, acting on Leydig cells [92]. In fact, Ocn-deficient male mice show reduced levels of testosterone, testis size and fertility [92].

Men demonstrate a correlation between age and bone loss which is apparent even though it is less marked compared to the one occurring in women [93]. In fact, aging men present bone loss in both trabecular and cortical compartments with increased cortical porosity [94,95], thus increasing the risk of fracture after the age of 70 [96]. As for women, male age-related bone loss is due to decreased circulating sex steroid hormones, necessary for bone growth and maintenance [97,98,99,100]. Furthermore, the possible correlation between androgen deficiency and metabolic syndrome (MetS) deserves further attention [101,102] as it is not yet fully elucidated. Several studies have shown the beneficial effects of testosterone replacement on bone and fat mass in hypogonadic men [103,104] confirming the necessity of filling this lack of knowledge. 

Systemic inflammation due to several conditions such as aging, insulin resistance/metabolic syndrome/diabetes and sexual hormone deficiency appears to impair the balance of body metabolism leading to bone loss. The pathological process characterized by the up-regulation of the inflammatory response that occurs with advancing age due to the elevation of the main inflammatory cytokines like interleukin IL-1, IL-6 and Tumor Necrosis Factor-alpha (TNF-α) has been recently named “inflammaging” [105]. This process is mainly due to reduced gonadal hormone levels and aging, conditions leading to the characteristic increase of catabolic cytokines shown in the elderly [106]. The molecular action of TNFα in bone resorption is in large measure a consequence of its ability to stimulate activation of the Nuclear Factor kappa-B (NF-κB) transcription factor. This pathway is also a great mediator of Receptor activator of nuclear factor kappa-B ligand (RANKL)-induced signal transduction, and not surprisingly TNFα potently augments RANKL-induced osteoclast formation. In fact, RANKL, a member of the TNF cytokine family, has a crucial role in the differentiation of osteoclast precursors into activated osteoclasts, and it is up-regulated during the inflammatory response [107]. Confirming that inflammation is itself capable of jeopardizing bone health, it has been demonstrated that inflammatory systemic conditions such as Crohn’s disease and rheumatoid arthritis are associated with reduced BMD, osteoporosis and fragility fractures [108].

It is well established that obese subjects have lower serum levels of adiponectin compared to normal-weight individuals, and its levels increase after weight loss [109,110]. Adiponectin serum levels are inversely correlated with insulin resistance [111]. However, the effects of adiponectin on bone health remain controversial. Adiponectin activity favors osteoblastogenesis and inhibits osteoclast formation *in vitro*, potentially contributing to an increase in bone mass [112]. In contrast, adiponectin knock-out mice show increased bone density, suggesting an indirect effect of adiponectin on bone tissue, possibly through modulation of circulating growth factor activity or insulin sensitivity [113]. For example, this adipokine decreases circulating insulin levels, reducing its anabolic effect, which in turn might inhibit bone growth [114]. Several authors have shown an inverse correlation between serum adiponectin and BMD in both women and men [115,116,117]. Other authors, in an Italian population of 600 elder men and postmenopausal women, have failed to confirm such a finding in men while confirming it in women [118]. Tamura *et al.* showed instead a positive correlation with BMD (evaluated in distal radius) in patients with type 2 diabetes [119]. Given the controversial evidence currently available, further studies are warranted to understand whether the characteristically low adiponectin levels in obese subjects are protective or detrimental with regards to bone health.

Leptin is an adipokine that decreases appetite and increases energy expenditure in malnutrition, and circulates at higher levels in obese subjects compared with normal-weight ones. Both negative and positive correlations between leptin and BMD have been described in humans [120,121]; in fact, while leptin seems to promote the differentiation of osteoblasts [122], it also seems to inhibit bone formation acting through the sympathetic nervous system and cocaine-amphetamine regulated transcript [123]. In peri-and postmenopausal women a positive correlation between leptin and BMD and a negative correlation with markers of bone resorption have been observed (dependent on BMI and fat content) [124]. The above correlations are weaker in postmenopausal women with osteoporosis, in comparison with healthy women in the same age group [125]. In obese postmenopausal women the correlations between leptin and BMD and bone turnover markers are stronger (mainly for bone resorption markers) than in lean women in the same age group [126]. Leptin resistance in the central nervous system may explain the previous assumption, in fact an imbalance between leptin levels in serum and cerebrospinal fluid is present in obese subjects (leptin cerebrospinal fluid levels are much lower than serum leptin levels in obese subjects compared with normal weight ones) [125,127].

High leptin levels in obese individuals can have a protective effect on bone tissue due to the interaction between leptin and the RANKL/ RANK/Osteoprotegerin system. It was proposed that the beneficial effect of leptin on bone metabolism was a result of the inhibition of the receptor activator of NF-κB ligand and the improved expression of osteoprotegerin [128].

Ghrelin is a gut-derived hormone, which increases food intake in both rodents [129,130] and humans [131], and decreases metabolic rate [132] and fat catabolism [133]. Ghrelin also appears to be involved in bone metabolism *via* modulation of osteoblast differentiation and function [134]. Although some *in vitro* findings suggest that ghrelin has protective effects on bone health, the available data are controversial. Napoli *et al.* have recently shown that ghrelin is associated with trabecular BMD but not with total or cortical BMD in post-menopausal women [118].

Traditionally, bone marrow fat function has always been conceived as a physical support [135]. However, it has been recently reported that its role is far more complex and active, appearing to be directly implicated in bone metabolism [136,137,138]. As mentioned above, both osteoblasts and adipocyte progenitors have roots in a common mesenchimal progenitor, whose ability in differentiating into both lineages is impaired in some conditions, such as obesity, where adipogenesis becomes the preferential pathway [136,137]. Moreover, it has been reported that bone marrow fat inversely relates to bone strength [139]. A study in obese young men and women conducted by Miriam *et al.* has recently shown a strong correlation between several lipid parameters such as serum triglyceride, intrahepatic and intra-myocellular lipids and bone marrow fat, maintaining statistical significance even when controlled for potential confounders like BMI, age, level of physical activity and serum insulin levels. Moreover, HDL levels were found to be inversely related to marrow fat content. As bone marrow adiposity is known to be inversely correlated to BMD, the authors suggested that ectopic and serum lipid levels are modulated by the same factors as bone marrow fat and may be potentially detrimental to bone health [140].

The role of lipid and lipoprotein oxidation in the pathophysiology of osteoporosis has been suggested by several studies [141,142]. In a recent study on mice fed an atherogenic high fat diet, it was reported that T-lymphocytes may have a role in the hyperlipidemia-induced bone loss. In fact, in this study, it was demonstrated that T-lymphocytes isolated from the spleen and bone marrow from the high-fat group showed increased expression of RANKL and not only became hyperlipidemic but also showed significantly reduced mineral content. T-lymphocytes from the high fat group tested *ex vivo* showed an increased expression of IL-6, TNF-alpha, IL-1beta and INF-gamma, cytokines that have a well-documented association with inflammation and bone loss.

Several potential mechanisms have been suggested to elucidate the complex relationship between bone and adipose tissue. The endocrine system, adipokines and inflammation have been proposed as some of the components of such interplay. Fat tissue is one of the major sources of aromatase that has a crucial role in the maintenance of skeletal health. Several adipokines, such as leptin and adiponectin, have shown a direct effect on bone metabolism. An inflammation marker as TNFα potently augments RANKL-induced osteoclast formation. However, the effects of these factors on bone health remain controversial, especially because some of them presented both potential positive and negative impacts. More studies are needed to elucidate this complex relationship.

## 5. Environmental Factors

A complicated interaction between behavioral, genetic, and environmental factors account for the obesity and osteoporosis epidemic. Although there is a strong genetic component to both conditions, given their abrupt prevalence increase, these cannot be due solely to genetic causes, and must also be caused by changes in the environment.

It is clear that diet and physical activity are the primary modifiable factors associated with obesity and bone health. Evidence from animal studies prove that over-nutrition and consequent obesity increase fracture risk by direct and indirect effects on bone and calcium absorption [143,144]. It has been shown that rodents BMD and bone quality are impaired when an “obesiogenic” diet is administered during growth [145,146,147]. A high-fat diet (HFD) resulted in greater lean and fat mass and lower cortical bone biomechanical properties when compared to low-fat diet but these effects vary depending on age [148]. HFD appears to affect bone remodeling leading to decreased femoral trabecular bone mass [147]. Excessive fat and sucrose intake impair bone geometry and mechanical properties of cortical bone in mice [149] and these effects are exacerbated after long term dietary exposure [150]. The excessive intake of sugars, such as fructose or glucose, has been shown to impair BMD, BMC and mechanical strength in rats [151,152,153]. Protein sources during excessive energy intake may also influence bone response. Skim milk intake improves trabecular bone architecture in obese rats on high fat and high sucrose diet to a greater extent than either whey protein or casein alone [154]. Despite evidence from animal models, there is little data from prospective studies or RCT conducted on human beings about the effects of macronutrients on bone. Some studies evaluated the effect of the long-chain ω-3 fatty acids, docosahexaenoic acid (DHA) and eicosapentaenoic acid on bone [155,156,157,158]. The majority of these studies have been conducted in adults and the findings are equivocal with respect to improvements in bone mass [159]. For these reasons, the National Osteoporosis Foundation assigned an inadequate level of evidence for the benefit of fat on bone [160]. Also, data regarding dietary proteins and bone quality mainly come from adult studies. The majority of prospective [161,162,163,164] and cross-sectional [165,166,167] studies support a positive relationship between protein intake and bone. As prospective studies and RCTs in children and adolescents are lacking, the National Osteoporosis Foundation conclude that there is a limited level of evidence for the benefit of protein on bone [160].

Regarding micronutrients, it is known that calcium supplementation has a beneficial effect on the bone, and there is a high level of evidence [160]. It is suggested that low calcium intake during early life may contribute to the later development of obesity and some of its co-morbidities [168]. It has also been shown that consumption of a rich source of calcium such as milk, besides increasing bone mass and inhibiting bone loss, reduces obesity risk in children [169].

On the other hand, weight loss is associated with 1%–2% bone loss at the hip and at highly trabecular sites, such as the trochanter and radius [170,171,172,173]. Age and initial body weight before caloric restriction appear not only to influence bone loss but also the anatomical sites, compartments and geometry of bone [174,175,176]. However, adequate dairy intake during weight loss resulted in higher lumbar spine BMD and Ocn compared to low dairy intake [177]. A weight-loss intervention program based on diet conducted on overweight and obese individuals induced a small decrease in total hip BMD, but not lumbar spine BMD. The decrease was small when compared to the well-known metabolic advantages of a lower BMI [178]. More recently, it has been shown that moderate weight loss in overweight and obese men did not decrease BMD at any anatomical site or alter cortical and trabecular bone and geometry [179].

Adding exercise to dietary-induced weight loss may reduce bone damage by decreasing mechanical stress [180]. In fact, exercise training added to weight-loss therapy among obese older adults not only reduces frailty but also appears to ameliorate weight loss-induced reduction in bone mineral density (BMD) and lean body mass [181,182]. RCTs suggest that exercise such as high intensity resistance training [183] or a combined aerobic and resistance training program [182] is effective in maintaining total body [183] and regional [182] bone mass in overweight and obese older adults undergoing intentional weight loss. Reid *et al.* found an inverse relationship between bone mass and body fat content in subjects with high physical activity [184]. In a large cohort of postmenopausal women with abdominal obesity, those in the highest (≥0.90) *vs.* lowest (<0.75) category of waist-to-hip ratio had increased risk of hip fracture if their activity was less than the population median [185]. Hence, physical activity should consolidate a thorough weight loss program in obese older adults in order to minimize the adverse effects of weight loss on bone health.

Although diet and physical activity are the primary variables that explain the obesity and osteoporosis epidemic, other factors are now being considered as contributors.

Endocrine disruptors (ED) are “exogenous agents that interfere with the production, release, transport, metabolism, binding, action or elimination of the natural hormones in the body responsible for the maintenance of homeostasis and the regulation of developmental processes” (U.S. Environmental Protection Agency Endocrine Disruptors Research [186]. The group of molecules identified as ED is highly heterogeneous and includes heavy metals such as cadmium and lead and several synthetic chemicals generally adopted in the solvents and lubricants industry. They may also be found in plastic compounds, plasticizers, pharmaceutical agents and pesticides. Evidence from epidemiological and animal-based studies indicates that exposure to these chemicals *in utero* and during early life may result in birth defects, behavioral disorders and cancer [187]. In 2011, the National Toxicology Program sponsored a workshop whose aim was to review environmental substances that may be implied in the obesity epidemics. The workshop also supported the “developmental obesogen” hypothesis, which suggests that chemical exposures may alter neural development that regulates feeding behavior later in life and predispose some individuals to gain weight despite their efforts to limit caloric intake and increase levels of physical activity [188]. Bone is an endocrine target tissue highly sensitive to numerous ED [189]. Data from *in vitro* and *in vivo* studies indicate that tributyltin chloride (TBT) can disrupt the process of bone deposition and remodeling [190,191,192]. Similarly, TBT seems to stimulate adipogenesis and ectopic adipocyte formation through PPAR-γ activation [193,194]. 

Finally, obesity seems to enhance a negative effect of ED on bone. In fact, a recent study has shown a significant association between blood cadmium levels and osteoporosis in obese males compared to non-obese ones. The authors hypothesized that simultaneous exposure to cadmium and obesity-induced inflammatory state lead to impaired bone formation due to oxidative stress [195].

Environmental factors are responsible for the increased incidence of obesity and osteoporosis and play an important role in the cross-talk between these two conditions. Both over-nutrition, with consequent obesity, and weight loss are associated with qualitative and/or quantitative bone tissue alterations. Advice for weight reduction and/or lifestyle changes with the aim of reducing obesity related comorbidities needs to be encouraged, but it should be balanced with proper exercise and adequate calcium intake to prevent osteoporosis.

Very limited studies, published mostly within the last few decades, indicate that bone and adipose tissues are negatively affected by exposure to persistent ED. The mechanisms behind the deleterious effects of ED on these tissues need further evaluation.

## 6. Anti-Obesity Drugs and Bone Metabolism

The incretin system includes a large family of gastrointestinal hormones, most of their physiological effects being achieved by Glucose-dependent Insulinotropic Peptide (GIP) and glucagon-like peptide-1 (GLP-1) [196]. The effect of these peptides consists in reducing blood glucose levels by inhibiting glucagon release, decreasing gastric emptying and food intake and potentially enhancing insulin secretion from beta cells. GIP is secreted by K-cells in the proximal regions of the small intestine (duodenum and proximal jejunum) [197], whereas GLP-1 is produced by L-cells, localized primarily in the distal ileum and colon [198]. During post-prandial phase, gut endocrine cells release GIP and GLP-1 in response to nutrient assumption [199]. Physiologically, GIP and GLP-1 are quickly degraded by dipeptidyl peptidase-4 (DPP-4). Instead, GLP-1 receptor analogues, such as liraglutide, used as treatment for diabetes, are resistant to DPP-4 degradation resulting in extended half-life. Furthermore, liraglutide has been recently approved as a treatment to reduce body weight in non-diabetic patients. It has been observed that incretins influence bone metabolism in several ways. Incretins may regulate cellular proliferation of progenitor bone forming mesenchymal cells [200]. Furthermore, GLP-1, through GPI/IPG-coupled receptor, is able to interact with osteoblasts [201], stimulating osteoblast proliferation [198] and enhancing collagen type I expression and ALP activity [202]. GLP-1 administration, or its analogue enzyme exendin-4, has resulted in increased trabecular bone mass in diabetic rats [203,204,205], but also in non-diabetic osteoporotic OVX rats [206,207]. Contrasting data has been collected relating to the effects of GLP-1 or GLP-2 analogue therapy on BMD in human subjects. In post-menopausal women treated with GLP-2, it has been observed a dose-dependent increase in total hip BMD [208], whereas after exenatide treatment, compared to insulin glargine, no differences in terms of BMD have been found in metformin-treated patients [209]. Recently, Gilbert *et al.* have investigated the effect of liraglutide treatment, and after 2 years no detrimental effect on BMD in diabetic post-menopausal women have been observed [210]. In a recent study, it has been found that treatment with a long-acting GLP-1 analogue prevented bone loss after a weight reduction due to a low-calorie diet compared to low-calorie diet al.one. Furthermore, after treatment with GLP-1 analogue, bone formation markers, such as P1NP, have increased [211]. Two meta-analysis have investigated the effects of GLP-1 agonists on fracture risk.

Mabilleau and colleagues found that GLP-1 agonists do not affect fracture risk [212]. However, the total number of fractures reported was only 19 (GLP-1 agonist, 13; comparator, 6) [212]. Recently, Su *et al.* have observed that different GLP-1 analogues showed different fracture risks. Specifically, liraglutide has been associated with a significant decrease in fracture risk (MH-OR = 0.38, 95% CI 0.17–0.87); on the other hand, exenatide was correlated with more fracture events (MH-OR = 2.09, 95% CI 1.03–4.21) [213].

Orlistat is an inhibitor of intestinal lipases, resulting in reduced intake of lipids through the bowel. Very few studies have investigated the effect of Orlistat on bone metabolism and the authors have not found any effect on bone mass [214,215]. However, Gotfredsen *et al.* observed a malabsorption of vitamin D and calcium and suggested an increased bone turnover [215].

In summary, few studies investigated the effects of GLP-1 analogues and orlistat on bone metabolism. Regarding GLP-1 analogues, the available evidence seems to indicate that they do not have any detrimental effect on bone health. To the other hand, Orlistat seems not to have any effect on bone metabolism, but more studies should be conducted to investigate its impact on bone metabolism.

## 7. Weight Reduction and Bone Health. Is It Actually Worthwhile?

Given the controversial association between obesity and bone health, the question is whether weight loss is beneficial or unsafe for bone quality and density. Bone mobilization and decrease of mineral content and density are generally associated with weight loss, either obtained with nutritional intervention or bariatric surgery [216,217].

Several factors can influence the risk of bone loss such as initial body weight, age, gender, level of physical activity and conditions of dieting like the extent of energy restriction and specific levels of nutrients intake. The bone response to weight reduction varies among different populations. Weight loss in miscellaneous populations including pre, peri and post-menopausal women, and/or men leads to a loss of total body bone mineral density (BMD; 0%–2.5%) and content (BMC; 3%–4%) as well as variable losses at regional bone sites (1%–13%) [216,218,219]. In more homogenous populations, studies have shown more consistent findings. For example, in postmenopausal women a 4%–13% weight reduction led to 1%–4% bone loss and a rise in bone turnover compared with a weight-stable group [170,220,221,222]. Older overweight or lean women close to menopause (mean age 48 years old) responded to a moderate weight reduction (5%) in a similar manner to that described for postmenopausal women, showing some bone loss (0.8% at the hip) [170]. Weight loss studies in premenopausal women (mean age of 45 years old) showed either a small decrease in total body and regional BMD and BMC of 0.5%–1.8% [223,224], or no bone changes in controlled trials [225,226]. In an interventional trial with middle-aged men, moderate weight loss (7%) caused a 1% bone loss [227]. Epidemiologic studies of elderly men (mean: 70 years of age) demonstrated that weight loss is an important predictor of BMD decrease [172] and leads to an increased incidence of osteoporosis [228]. 

It can be speculated that greater weight loss (average 14%) during a relatively short period of time (3–4 months) results in significant bone weakening [224,229], while a more modest weight loss over a longer period of time (6 months) results in little (1%) [223] or no bone loss [225,226] at least in premenopausal women. 

Weight regain is associated with regain of bone in pre- [216,230] but not in post-menopausal women [231] suggesting that the endocrine system of older age does not support bone growth in the context of positive energy imbalance [232].

As mentioned before, physical activity is a crucial factor in the weight loss-bone health correlation. Villareal *et al.* have demonstrated that weight loss induces bone weakening which is significantly prevented by exercise training in obese elderly (>65 years) individuals [181]. However, the mechanism for this observation remains unclear. Sclerostin, an inhibitor of bone formation, increases in states of unloading and may act in the weight loss related bone alterations. Physical activity may partially help reducing the negative effect of weight loss on bone metabolism preventing weight loss related sclerostin increase in elderly individuals [233]. Exercise training as an add-on to weight loss therapy prevents bone turnover markers increase and hip BMD reduction in obese subjects [182].

There is not enough evidence in literature about the influence of weight loss in children on bone mass and quality to allow generalizations on this population. Although it seems to be established that bone loss occurs with weight loss in older women and perhaps in older men, it remains unclear whether there this effect could be applied to younger individuals or children with weight reduction [234].

In summary, a great deal of evidence has suggested that weight loss is generally associated with a decrease of mineral content and density. However, recent findings appear to show that a combination of weight loss and exercise training significantly prevents this weight loss related effect in obese older adults. This could be an important consideration in establishing an appropriate treatment in this population. However, the mechanisms underlying such observations remains unclear and further studies are needed to clarify this effect.

## 8. Conclusions

Available studies have provided contrasting findings: some authors suggest that obesity has detrimental effects on bone health, while others have revealed its potential protective role. Regardless, a “U” shape relationship seems to exist between BMI and fracture risk. Indeed, the higher the BMI, the lower the protection of weight on bone. Mostly, abdominal/visceral obesity is associated with lower BMD. In particular, systemic inflammation due to several conditions such as aging, insulin resistance/metabolic syndrome/diabetes and sexual hormone deficiency appear to impair the balance of body composition leading to bone loss.

What might be considered is to add markers of metabolic health, such as waist circumference, fasting plasma glucose, lipid and C-reactive protein serum concentrations to identify obese subjects with higher risk of fracture.

It is important to pay attention to lifestyle modifications and/or treatments that may lead to plentiful and fast weight loss because it may be associated with a significant bone mass loss. This loss may be limited by associating the diet to proper exercise and adequate calcium intake.

Unfortunately, obesity is a complex disease of multifactorial aetiology, with its own pathophysiologies and comorbidities such as diabetes mellitus that can lead to bone fragility. Although *in vitro* studies are able to investigate the effect of each adipokine on bone metabolism, it is very hard to study how obesity can affect bone system *in vivo*.

As obesity increases fracture risk independently of BMD, *in vivo* bone material properties assessment might represent a useful tool to provide more information about the risk of fracture in these kind of subjects [235].

Furthermore, most of the studies that have investigated the effect of obesity on bone health in human subjects are observational ones; therefore, they can suggest but not demonstrate the potential correlation between obesity and bone metabolism.

Larger and robust pre-clinical and clinical randomized control trials are needed to better understand the relation between obesity and bone health.

## Figures and Tables

**Table 1:** Cross-sectional studies and case-control designs focused on the relation between Obesity and bone health in humans.

Author, Year	Country	Type of Study	Subjects	RR/OR (95% CI)	Results
Michel BA, 1988 [28]	U.S.	Cross-sectional study	78 healthy subjects, ≥50 years	-	Moderate weight bearing exercise may increase lumbar bone density. *Comment of the author:* maybe, extremely vigorous exercise could be detrimental to bone density in individuals after age 50
Haffner SM, 1993 [31]	U.S.	Cross-sectional study	317 premenopausal and postmenopausal women	-	Lumbar spine and femoral neck density were positively correlated with BMI. The same between femoral neck density with fasting insulin level in younger women after adjustment for age (r = 0.214, *p* < 0.01). After adjustment for BMI, femoral neck density was not significantly correlated with fasting insulin level (*p* = 0.08). Adjustment for glucose and insulin levels does not explain the linkage between bone density and obesity
DiPietro L, 1993 [57]	U.S.	Cross-sectional study	2285 postmenopausal women, aged 50–77 years	Baseline body mass index in the highest quartile (>37 kg/m^2^) experienced a 70% lower rate of hip fracture compared with women in the lowest quartile (28.7 kg/m^2^) (RR = 0.32; 95% CI 0.12–0.82)	Although reported education level, physical activity level, smoking history and estrogen replacement were significantly (*p* < 0.0001) associated with BMI, these covariates were not related to hip fracture in the multivariable analysis
Albala C, 1996 [29]	Chile	Case-control study	113 obese and 50 non-obese postmenopausal women	In Obese women, a decreased risk of osteopenia in femoral neck (Age adjusted OR = 0.36, 0.17–0.75); lumbar spine (Age adjusted OR = 0.43, 0.20–0.91)	Obese women showed a higher BMD; obesity exerts protection due to a decreased SHBG thus increasing free sex steroids. Hyperinsulinemia may produce a decrease in the production of IGFBG-1, leading to an increase of IGF-1, that could stimulate the proliferation of osteoblasts
Manzoni P, 1996 [38]	Italy	Cross-sectional study	65 obese and 50 normal-weight children and adolescents (age range: 5–18 years relative body weight: 160% ± 23% and 101% ± 12%, respectively)	-	No differences in TBMC and RBMC among obese and normal-weight children groups, after correction for the confounding variables age and sex
Goulding A, 1998 [30]	New Zealand	Cross-sectional study	54 postmenopausal women	-	No evidence for an association between plasma levels of leptin and biochemical markers of either osteoclastic or osteoblastic activity
Kanis J, 1999 [34]	UK	Case-control study	730 men with hip fracture, 1132 age-stratified controls, 50 years or more.	The effect of BMI on risk was linear, with a change for each unit of BMI of 6.8% (95% CI 4%–9%)	A low BMI was associated with a significantly increased risk of hip fracture in a dose-dependent manner
Fischer S, 2000 [39]	Chile	Cross-sectional, case control study	16 obese children (8 male, 8 female) aged 5 to 13 years. 16 healthy eutrophic children matched for sex, chronological age, height, and pubertal stage were enrolled as controls	-	Obese children have more total body BMC than eutrophic children. No significant difference was showed in regional hip BMD and lumbar spine BMD in the group of obese and normal children
Goulding A, 2000 [42]		Cross-sectional study	200 girls and 136 boys, aged 3–19 years	-	Girls and boys (in overweight and obese) showed a mismatch between body weight and bone development during growth: their bone mass and bone area are low for their body weight
van der Voort DJ, 2001 [59]	The Netherlands	Cross-sectional study	4725 postmenopausal women, 50–80 years of age	BMI > 30 kg/m^2^ and fractures elsewhere: OR 1.4 (1.0–1.9).	Women with normal BMD showed statistically significant lower fracture risk than osteoporotic women. Women with a possibly decreased BMI were most often osteoporotic and had sustained more fractures during the past 5 years’ than expected. Women who had (probably) always been obese were less often osteoporotic and had a much lower fracture risk
Goulding A, 2002 [43]	New Zealand	Cross-sectional study	202 boys and 160 girls, aged 3–19 years	Overweight and obese groups were 0.92 (95% CI 0.87–0.97) and 0.88 (95% CI 0.80–0.96) for girls and 0.96 (95% CI 0.91–1.02, NS) and 0.87 (95% CI 0.78–0.96) for boys, respectively	During growth children (in overweight and obese) do not increase their spinal BMC due to a compensation for their excessive weight
Davidson PL, 2003 [48]	New Zealand	Cross-sectional study	50 boys (25 obese pair-matched with 25 non-obese subjects), aged 4–17 years	-	Environmental modifications are unlikely to lower the risk of arm fracture in obese children to the same levels showed by non-obese children
Taylor ED, 2006 [47]	U.S.	Cross-sectional study	227 overweight and 128 nonoverweight children and adolescents	The prevalence of documented skeletal fractures in overweight than in nonoverweight children and adolescents (odds ratio (OR): 4.54; 95% confidence interval (CI): 1.6–13.2 *p* = 0.0053)	Fractures, impaired mobility, musculoskeletal difficulties, and lower extremity malalignment were more prevalent in overweight than nonoverweight children and adolescents
Sharma S, 2008 [33]	UK	Cross-sectional study	2035 men aged over 50 years	-	A low BMI, showed significantly, more hip fractures than those with fractures elsewhere
Gnudi S, 2009 [36]	Italy	Cross-sectional study	2235 postmenopausal women including those with fragility fractures of the hip (187), ankle (108), wrist (226) and humerus (85)	BMI had a protective effect against hip fracture: OR 0.949 (0.900–0.999); higher risk of humerus fracture: OR 1.077 (1.017–1.141)	Decreasing BMI increases the risk for hip fracture, whereas increasing BMI increases the risk for humerus fractures
Bredella MA, 2011 [50]	U.S.	Cross-sectional study	68 healthy obese premenopausal women	-	VAT exerts detrimental effects, whereas muscle mass exerts positive effects on BMD in premenopausal obese women. IGF-1 could be a mediator of the bad effects of VAT on bone health through effects on bone formation
Prieto-Alhambra D, 2012 [62]	Spain	Cross-sectional study	832,775 women aged ≥50 years were categorized into underweight/normal (*n*: 302,414), overweight (*n*: 266,798), and obese (*n*: 263,563)	Hip fractures were significantly less common in overweight and obese women than in normal/underweight women (rate ratio (RR) 0.77 (95% confidence interval (CI) 0.68 to 0.88), RR 0.63 (95% CI 0.64–0.79), *p* < 0.001, respectively). Pelvis fracture rates were lower in the overweight (RR 0.78 (95% CI 0.63–0.96), *p* = 0.017) and obese (RR 0.58 (95% CI 0.47–0.73), *p* < 0.001) groups. Conversely, obese women were at significantly higher risk of proximal humerus fracture than the normal/underweight group (RR 1.28 (95% CI 1.04–1.58), *p* = 0.018)	An age-related increase in incidence was showed for all BMI groups at all fracture sites; obese women with hip, clinical spine and pelvis fracture were significantly younger at the time of fracture than normal/underweight women, whereas those with wrist fracture were significantly older. The association between obesity and fracture in postmenopausal women is site-dependent, obesity being protective against hip and pelvis fractures but associated with an almost 30% increase in risk for proximal humerus fractures when compared with normal/underweight women
Kessler J, 2013 [45]	U.S.	Cross-sectional study	Electronic medical records of 913,178 patients, aged 2 to 19 years	Overweight, moderately obese, and extremely obese patients all had an increased OR of fractures of the foot (1.14, 1.23, and 1.42, respectively, (1.04–1.24, 1.12–1.35, and 1.26–1.61), respectively- along with the ankle, knee, and leg (1.27, 1.28, and 1.51, respectively, with 1.16–1.39, 1.15–1.42, and 1.33–1.72, respectively)	Increasing BMI is associated with increased odds of foot, leg, ankle and knee fractures in children
Cohen A, 2013 [49]	U.S.	Cross-sectional study	40 healthy premenopausal women	-	At the tissue level, premenopausal women with more central adiposity showed inferior bone quality and stiffness and markedly lower bone formation
Correa Rodriguez M, 2014 [40]	Spain	Cross-sectional study	157 adolescents (93 women and 64 men) Mean age: 14.22 ± 1.41 year	-	BMD increases in response to increased muscle mass in adolescents with overweight and/or obesity
Jeddi M, 2015 [41]	Iran	Cross-sectional study	472 subjects (235 girls, 237 boys) aged 9–18 years	-	Lean mass was the main predictor of BMD in both genders. Physical activity appears to positively impact on lean mass
Shen J, 2015 [53]	U.S.	Cross-sectional Study	672 men (mean age: 73 years)	Obese men were 4 times more likely to have aload-to-strength ratio >1.0 compared to normal-weight men (OR: 4.66; 95% CI 2.16–10.05; *p* < 0.0001).	About non-obese men (BMI < 30), increasing BMI was associated with higher integral, cortical and trabecular BMD, integral volume, cross-sectional area, and percent cortical volume (all *p* < 0.001). About obese men (BMI ≥ 30), increasing BMI was not associated with any of those parameters. Furthermore, compared to non-obese men, obese men had a higher hip strength, but also a higher ratio of impact force to strength (*p* < 0.0001), in theory increasing their risk of hip fracture despite their increased strength

**Table 2:** Cohort studies focused on the relation between obesity and bone health in humans.

Author, Year	Country	Type of Study	Subjects	RR/OR (95% CI)	Results
Joakimsen RM, 1998 [58]	Norway	Cohort study	12,270 (922 persons with fractures) middle-aged	Change in body mass index was not associated with fractures among men, except for a lower incidence of hip fractures (not only low-energy) among those who had gained weight (RR 0.69, 95% CI 0.50–0.95, age adjusted per unit BMI increase). Women who had increased their body mass index had a lower risk of all low-energy fractures (RR 0.95, 95% CI 0.90–1.01, age adjusted per unit BMI increase) and of low-energy fractures in the lower extremities (RR 0.88, 95% CI 0.80–0.97, age adjusted per unitBMI increase)	High body height is a risk factor for fractures, and 1 in 4 low-energy fractures among women today could be ascribed to the increase in average stature since the turn of the century. Low BMI was associated with a higher risk of fractures
Honkanen RJ, 2000 [60]	Finland	Cohort study	11,798 women. Mean baseline age of these women was 52.3 (SD 2.9) years (range 47–56 years) and 68% were postmenopausal	Overweight (BMI > 25 kg/m^2^) decreased the perimenopausal distal forearm fracture by 36% (*p* = 0.0002)	Overweight protects against perimenopausal distal forearm fracture
Holmberg AH, 2006 [32]	Sweden	Cohort study	22,444 men and 10,902 women, mean age 44 and 50 years	High BMI and forearm fractures (RR 0.88, 95% CI 0.81–0.96) High BMI and risk of proximal humerus and ankle fractures (RR 1.21–1.33). High BMI and forearm fractures (RR 0.88, 95% CI 0.81–0.96)	High BMI significantly increased the risk of proximal humerus and ankle fractures while, by contrast, lowering the risk of forearm fractures
Wetzsteon RJ, 2008 [44]	U.S.	Cohort study	302 children healthy weight and 143 children overweight, (9–11 years)	-	Bone strength did not adapt to excess body fat. Rather, bone strength was adapted to the greater muscle area in overweight group of children.
Lee SH, 2010 [35]	Korea	Cohort study	9351 subjects (4732 men and 4619 women) aged 40 to 69 years were followed for a mean of 46.3 ± 2.2 months	In women, Obesity and risk of fracture 1.29 (0.76–2.18)	Older age, lower BMI, and previous fracture history were positively associated with fracture risk in men and women
Premaor MO, 2013 [52]	Brazil	Cohort study	139,419 men: underweight/normal (*n* = 26,298), overweight (*n* = 70,851), and obese (*n* = 42,270), ≥65 years	A statistically significant reduction in clinical spine and hip fractures was observed in obese (relative risk (RR), 0.65; 95% confidence interval (CI), 0.53–0.80 and RR, 0.63; 95% CI 0.54–0.74, respectively), and overweight men (RR, 0.77; 95% CI 0.64–0.92 and RR, 0.63; 95% CI 0.55–0.72, respectively) when compared with underweight/normal men. Additionally, obese men had significantly fewer wrist/forearm (RR, 0.77; 95% CI 0.61–0.97) and pelvic (RR, 0.44; 95% CI 0.28–0.70) fractures than underweight/normal men. Conversely, multiple rib fractures were more frequent in overweight (RR, 3.42; 95% CI 1.03–11.37) and obese (RR, 3.96; 95% CI 1.16–13.52) men	Obesity was associated with a reduced risk of clinical spine, pelvis, hip, and wrist/forearm fracture and increased risk of multiple rib fractures when compared to normal or underweight men
Ishii S, 2014 [51]	Japan	Cohort study	1924 women, premenopausal or early perimenopausal	The relative increment in fracture hazard in obese women compared to normal weight women was also statistically significant: 78% (95% CI 13%–181%, p ¼ 0.01). In stark contrast, obesity was significantly associated with decreased fracture hazard when adjusted instead for any of the composite indices of femoral neck strength relative to load: relative decrement in fracture hazard in obese relative to low weight women was 57% (95% CI 24%–76%) after adjusting for CSI, 41% (95% CI 1%–65%) after adjusting for BSI, and 53% (95% CI 16%–74%) after adjusting for ISI	There are 3 major mechanisms by which obesity influences fracture risk: increased impact forces, increased BMD in response to greater skeletal loading, and greater absorption of impact forces by soft tissue padding
Søgaard AJ, 2015 [54]	Norway	Cohort study	19,918 women and 23,061 men, aged 60–79 years	Compared to women with a BMI of <22 kg·m^−2^, the HR for hip fracture was 0.76 (95% CI 0.65–0.89) in women with a BMI between 22 and 24 kg·m^−2^, 0.56 (95% CI 0.48–0.65) in women with a BMI between 25 and 29 kg·m^−2^, and 0.42 (95% CI 0.35–0.51) in women with a BMI ≥ 30 kg·m^−2^. In men, the corresponding HRs for hip fracture were 0.62 (95% CI 0.50–0.77), 0.49 (95% CI 0.40–0.60) and 0.49 (95% CI 0.37–0.63), respectively	Abdominal obesity was associated with an increased risk of hip fracture when body mass index was taken into account

## Abbreviations

The following abbreviations are used in this manuscript:

BMI	body mass index
BMD	bone mineral density
BMC	bone mineral content
SHBG	sex hormone binding globulin
CRFs	clinical risk factors
TBMC	body compartments on total BMC
RBMC	regional BMC
DFF	distal forearm fracture
HRT	ormone replacement therapy
CSI	Compression Strength Index
BSI	Bending Strength Index
ISI	Impact Strength Index

## References

[1] Greco E.A., Lenzi A., Migliaccio S. (2015). The obesity of bone. Ther. Adv. Endocrinol. Metab..

[2] Kado D.M., Huang M.-H., Karlamangla A.S., Barrett-Connor E., Greendale G.A. (2004). Hyperkyphotic posture predicts mortality in older community-dwelling men and women: A prospective study. J. Am. Geriatr. Soc..

[3] Rössner S. (2002). Obesity: The disease of the twenty-first century. Int. J. Obes. Relat. Metab. Disord..

[4] Hu F.B. (2003). Overweight and obesity in women: Health risks and consequences. J. Womens Health (Larchmt).

[5] WHO Study Group (2000). Obesity: Preventing and managing the global epidemic. Report of a WHO consultation. World Health Organ. Tech. Rep. Ser..

[6] Greco E.A., Fornari R., Rossi F., Santiemma V., Prossomariti G., Annoscia C., Aversa A., Brama M., Marini M., Donini L.M. (2010). Is obesity protective for osteoporosis? Evaluation of bone mineral density in individuals with high body mass index. Int. J. Clin. Pract..

[7] Kim K.-C., Shin D.-H., Lee S.-Y., Im J.-A., Duk-Chul L. (2010). Relation between obesity and bone mineral density and vertebral fractures in Korean postmenopausal women. Yonsei Med. J..

[8] Compston J.E., Flahive J., Hosmer D.W., Watts N.B., Siris E.S., Silverman S., Saaq K.G., Roux C., Rossini M., Pfeilschifter J. (2014). Relationship of weight, height, and body mass index with fracture risk at different sites in postmenopausal women: The Global Longitudinal Study of Osteoporosis in Women (GLOW). J. Bone Miner. Res..

[9] Fact sheet N°311. http://www.who.int/mediacentre/factsheets/fs311/en/.

[10] Cawley J., Meyerhoefer C. (2012). The medical care costs of obesity: An instrumental variables approach. J. Health Econ..

[11] Finkelstein E.A., Trogdon J.G., Cohen J.W., Dietz W. (2009). Annual medical spending attributable to obesity: Payer-and service-specific estimates. Health Aff. (Millwood).

[12] Cawley J., Rizzo J.A., Haas K. (2007). Occupation-specific absenteeism costs associated with obesity and morbid obesity. J. Occup. Environ. Med..

[13] Gates D.M., Succop P., Brehm B.J., Gillespie G.L., Sommers B.D. (2008). Obesity and presenteeism: The impact of body mass index on workplace productivity. J. Occup. Environ. Med..

[14] Müller-Riemenschneider F., Reinhold T., Berghöfer A., Willich S.N. (2008). Health-economic burden of obesity in Europe. Eur. J. Epidemiol..

[15] WHO Study Group (1994). Assessment of fracture risk and its application to screening for postmenopausal osteoporosis. Report of a WHO Study Group. World Health Organ. Tech. Rep. Ser..

[16] Cooper C., Campion G., Melton L.J. (1992). Hip fractures in the elderly: A world-wide projection. Osteoporos. Int..

[17] Shuler F.D., Conjeski J., Kendall D., Salava J. (2012). Understanding the burden of osteoporosis and use of the World Health Organization FRAX. Orthopedics.

[18] Elffors I., Allander E., Kanis J.A., Gullberg B., Johnell O., Dequeker J., Dilsen G., Gennari C., Lopes Vaz A.A., Lyritis G. (1994). The variable incidence of hip fracture in southern Europe: The MEDOS Study. Osteoporos. Int..

[19] Kanis J.A., Johnell O., de Laet C., Jonsson B., Oden A., Ogelsby A.K. (2002). International variations in hip fracture probabilities: Implications for risk assessment. J. Bone Miner. Res..

[20] Thomas P.A. (2007). Racial and ethnic differences in osteoporosis. J. Am. Acad. Orthop. Surg..

[21] Bone Health and Osteoporosis: A Report of the Surgeon General. http://www.ncbi.nlm.nih.gov/pubmed/?term=20945569.

[22] Reid I.R. (2010). Fat and bone. Arch. Biochem. Biophys..

[23] Guh D.P., Zhang W., Bansback N., Amarsi Z., Birmingham C.L., Anis A.H. (2009). The incidence of co-morbidities related to obesity and overweight: A systematic review and meta-analysis. BMC Public Health.

[24] Zhao L.-J., Jiang H., Papasian C.J., Maulik D., Drees B., Hamilton J., Deng H.-W. (2008). Correlation of obesity and osteoporosis: Effect of fat mass on the determination of osteoporosis. J. Bone Miner. Res..

[25] Compston J. (2013). Obesity and bone. Curr. Osteoporos. Rep..

[26] Nielson C.M., Srikanth P., Orwoll E.S. (2012). Obesity and fracture in men and women: An epidemiologic perspective. J. Bone Miner. Res..

[27] Liberati A., Altman D.G., Tetzlaff J., Mulrow C., Gøtzsche P.C., Ioannidis J.P.A., Clarke M., Devereaux P.J., Kleijnen J., Moher D. (2009). The PRISMA statement for reporting systematic reviews and meta-analyses of studies that evaluate health care interventions: Explanation and elaboration. J. Clin. Epidemiol..

[28] Michel B.A., Bloch D.A., Fries J.F. (1989). Weight-bearing exercise, overexercise, and lumbar bone density over age 50 years. Arch. Intern. Med..

[29] Albala C., Yáñez M., Devoto E., Sostin C., Zeballos L., Santos J.L. (1996). Obesity as a protective factor for postmenopausal osteoporosis. Int. J. Obes. Relat. Metab. Disord..

[30] Goulding A., Taylor R.W. (1998). Plasma leptin values in relation to bone mass and density and to dynamic biochemical markers of bone resorption and formation in postmenopausal women. Calcif. Tissue Int..

[31] Haffner S.M., Bauer R.L. (1993). The association of obesity and glucose and insulin concentrations with bone density in premenopausal and postmenopausal women. Metabolism.

[32] Holmberg A.H., Johnell O., Nilsson P.M., Nilsson J., Berglund G., Akesson K. (2006). Risk factors for fragility fracture in middle age. A prospective population-based study of 33,000 men and women. Osteoporos. Int..

[33] Sharma S., Fraser M., Lovell F., Reece A., McLellan A.R. (2008). Characteristics of males over 50 years who present with a fracture: Epidemiology and underlying risk factors. J. Bone Jt. Surg. Br..

[34] Kanis J., Johnell O., Gullberg B., Allander E., Elffors L., Ranstam J., Dequeker J., Dilsen G., Gennari C., Vaz A.L. (1999). Risk factors for hip fracture in men from southern Europe: The MEDOS study. Mediterranean Osteoporosis Study. Osteoporos. Int..

[35] Lee S.H., Khang Y.-H., Lim K.-H., Kim B.-J., Koh J.-M., Kim G.S., Kim H., Cho N.H. (2010). Clinical risk factors for osteoporotic fracture: A population-based prospective cohort study in Korea. J. Bone Miner. Res..

[36] Gnudi S., Sitta E., Lisi L. (2009). Relationship of body mass index with main limb fragility fractures in postmenopausal women. J. Bone Miner. Metab..

[37] De Laet C., Kanis J.A., Odén A., Johanson H., Johnell O., Delmas P., Eisman J.A., Kroger H., Fujiwara S., Garnero P. (2005). Body mass index as a predictor of fracture risk: A meta-analysis. Osteoporos. Int..

[38] Manzoni P., Brambilla P., Pietrobelli A., Beccaria L., Bianchessi A., Mora S., Chiumello G. (1996). Influence of body composition on bone mineral content in children and adolescents. Am. J. Clin. Nutr..

[39] Fischer S., Milinarsky A., Giadrosich V., Dib G., Arriagada M., Arinoviche R. (2000). X-ray absorptiometry of bone in obese and eutrophic children from Valparaiso, Chile. J. Rheumatol..

[40] Correa Rodríguez M., Rueda Medina B., González Jiménez E., Navarro Pérez C.F., Schmidt-RioValle J. (2014). The levels of bone mineralization are influenced by body composition in children and adolescents. Nutr. Hosp..

[41] Jeddi M., Dabbaghmanesh M.H., Ranjbar Omrani G., Ayatollahi S.M.T., Bagheri Z., Bakhshayeshkaram M. (2015). Relative importance of lean and fat mass on bone mineral density in Iranian children and adolescents. Int. J. Endocrinol. Metab..

[42] Goulding A., Taylor R.W., Jones I.E., McAuley K.A., Manning P.J., Williams S.M. (2000). Overweight and obese children have low bone mass and area for their weight. Int. J. Obes. Relat. Metab. Disord..

[43] Goulding A., Taylor R.W., Jones I.E., Manning P.J., Williams S.M. (2002). Spinal overload: A concern for obese children and adolescents?. Osteoporos. Int..

[44] Wetzsteon R.J., Petit M.A., Macdonald H.M., Hughes J.M., Beck T.J., McKay H.A. (2008). Bone structure and volumetric BMD in overweight children: A longitudinal study. J. Bone Miner. Res..

[45] Kessler J., Koebnick C., Smith N., Adams A. (2013). Childhood obesity is associated with increased risk of most lower extremity fractures. Clin. Orthop. Relat. Res..

[46] Paulis W.D., Silva S., Koes B.W., van Middelkoop M. (2014). Overweight and obesity are associated with musculoskeletal complaints as early as childhood: A systematic review. Obes. Rev..

[47] Taylor E.D., Theim K.R., Mirch M.C., Ghorbani S., Tanofsky-Kraff M., Adler-Wailes D.C., Brady S., Reynolds J.C., Calis K.A., Yanovski J.A. (2006). Orthopedic complications of overweight in children and adolescents. Pediatrics.

[48] Davidson P.L., Goulding A., Chalmers D.J. (2003). Biomechanical analysis of arm fracture in obese boys. J. Paediatr. Child Health.

[49] Cohen A., Dempster D.W., Recker R.R., Lappe J.M., Zhou H., Zwahlen A., Müller R., Zhao B., Guo X., Lang T. (2013). Abdominal fat is associated with lower bone formation and inferior bone quality in healthy premenopausal women: A transiliac bone biopsy study. J. Clin. Endocrinol. Metab..

[50] Bredella M.A., Torriani M., Ghomi R.H., Thomas B.J., Brick D.J., Gerweck A.V., Harrington L.M., Breggia A., Rosen C.J., Miller K.K. (2011). Determinants of bone mineral density in obese premenopausal women. Bone.

[51] Ishii S., Cauley J.A., Greendale G.A., Nielsen C., Karvonen-Gutierrez C., Ruppert K., Karlamangla A.S. (2014). Pleiotropic effects of obesity on fracture risk: The Study of Women’s Health Across the Nation. J. Bone Miner. Res..

[52] Premaor M.O., Compston J.E., Fina Avilés F., Pagès-Castellà A., Nogués X., Díez-Pérez A., Prieto-Alhambra D. (2013). The association between fracture site and obesity in men: A population-based cohort study. J. Bone Miner. Res..

[53] Shen J., Nielson C.M., Marshall L.M., Lee D.C., Keaveny T.M., Orwoll E.S. (2015). The association between BMI and QCT-derived proximal hip structure and strength in older men: A Cross-Sectional Study. J. Bone Miner. Res..

[54] Søgaard A.J., Holvik K., Omsland T.K., Tell G.S., Dahl C., Schei B., Falch J.A., Eisman J.A., Meyer H.E. (2015). Abdominal obesity increases the risk of hip fracture. A population-based study of 43,000 women and men aged 60–79 years followed for 8 years. Cohort of Norway. J. Intern. Med..

[55] Paganini-Hill A., Chao A., Ross R.K., Henderson B.E. (1991). Exercise and other factors in the prevention of hip fracture: The Leisure World study. Epidemiology.

[56] Cummings S.R., Nevitt M.C., Browner W.S., Stone K., Fox K.M., Ensrud K.E., Cauley J., Black D., Vogt T.M. (1995). Risk factors for hip fracture in white women. Study of Osteoporotic Fractures Research Group. N. Engl. J. Med..

[57] DiPietro L., Welch G.A., Davis D.R., Drane J.W., Macera C.A. (1993). Body mass and risk of hip fracture among a national cohort of postmenopausal white women: A reanalysis. Obes. Res..

[58] Joakimsen R.M., Fønnebø V., Magnus J.H., Tollan A., Søgaard A.J. (1998). The Tromsø Study: Body height, body mass index and fractures. Osteoporos. Int..

[59] Van der Voort D.J., Geusens P.P., Dinant G.J. (2001). Risk factors for osteoporosis related to their outcome: Fractures. Osteoporos. Int..

[60] Honkanen R.J., Honkanen K., Kröger H., Alhava E., Tuppurainen M., Saarikoski S. (2000). Risk factors for perimenopausal distal forearm fracture. Osteoporos. Int..

[61] Watts N.B. (2014). Insights from the Global Longitudinal Study of Osteoporosis in Women (GLOW). Nat. Rev. Endocrinol..

[62] Prieto-Alhambra D., Premaor M.O., Fina Avilés F., Hermosilla E., Martinez-Laguna D., Carbonell-Abella C., Nogués X., Compston J.E., Díez-Pérez A. (2012). The association between fracture and obesity is site-dependent: A population-based study in postmenopausal women. J. Bone Miner. Res..

[63] Johansson H., Kanis J.A., Odén A., McCloskey E., Chapurlat R.D., Christiansen C., Cummings S.R., Diez-Perez A., Eisman J.A., Fujiwara S. (2014). A meta-analysis of the association of fracture risk and body mass index in women. J. Bone Miner. Res..

[64] Takada I., Suzawa M., Kato S. (2005). Nuclear receptors as targets for drug development: Crosstalk between peroxisome proliferator-activated receptor gamma and cytokines in bone marrow-derived mesenchymal stem cells. J. Pharmacol. Sci..

[65] Moerman E.J., Teng K., Lipschitz D.A., Lecka-Czernik B. (2004). Aging activates adipogenic and suppresses osteogenic programs in mesenchymal marrow stroma/stem cells: The role of PPAR-gamma2 transcription factor and TGF-beta/BMP signaling pathways. Aging Cell.

[66] Pei L., Tontonoz P. (2004). Fat’s loss is bone’s gain. J. Clin. Investig..

[67] Vidal-Puig A.J., Considine R V., Jimenez-Liñan M., Werman A., Pories W.J., Caro J.F., Flier J.S. (1997). Peroxisome proliferator-activated receptor gene expression in human tissues. Effects of obesity, weight loss, and regulation by insulin and glucocorticoids. J. Clin. Investig..

[68] Kirkland J.L., Tchkonia T., Pirtskhalava T., Han J., Karagiannides I. (2002). Adipogenesis and aging: Does aging make fat go MAD?. Exp. Gerontol..

[69] Sepe A., Tchkonia T., Thomou T., Zamboni M., Kirkland J.L. (2011). Aging and regional differences in fat cell progenitors—A mini-review. Gerontology.

[70] Goodpaster B.H., Thaete F.L., Kelley D.E. (2000). Thigh adipose tissue distribution is associated with insulin resistance in obesity and in type 2 diabetes mellitus. Am. J. Clin. Nutr..

[71] Yim J.-E., Heshka S., Albu J., Heymsfield S., Kuznia P., Harris T., Gallagher D. (2007). Intermuscular adipose tissue rivals visceral adipose tissue in independent associations with cardiovascular risk. Int. J. Obes. (Lond.).

[72] Wronska A., Kmiec Z. (2012). Structural and biochemical characteristics of various white adipose tissue depots. Acta Physiol. (Oxf.).

[73] Ryan A.S., Nicklas B.J. (1999). Age-related changes in fat deposition in mid-thigh muscle in women: Relationships with metabolic cardiovascular disease risk factors. Int. J. Obes. Relat. Metab. Disord..

[74] Snijder M.B., Visser M., Dekker J.M., Goodpaster B.H., Harris T.B., Kritchevsky S.B., De Rekeneire N., Kanaya A.M., Newman A.B., Tylavsky F.A. (2005). Low subcutaneous thigh fat is a risk factor for unfavourable glucose and lipid levels, independently of high abdominal fat. The Health ABC Study. Diabetologia.

[75] Yim J.-E., Heshka S., Albu J.B., Heymsfield S., Gallagher D. (2008). Femoral-gluteal subcutaneous and intermuscular adipose tissues have independent and opposing relationships with CVD risk. J. Appl. Physiol..

[76] Cartier A., Côté M., Lemieux I., Pérusse L., Tremblay A., Bouchard C., Després J.-P. (2009). Age-related differences in inflammatory markers in men: Contribution of visceral adiposity. Metabolism.

[77] Addison O., LaStayo P.C., Dibble L.E., Marcus R.L. (2012). Inflammation, aging, and adiposity: Implications for physical therapists. J. Geriatr. Phys. Ther..

[78] Prior S.J., Joseph L.J., Brandauer J., Katzel L.I., Hagberg J.M., Ryan A.S. (2007). Reduction in midthigh low-density muscle with aerobic exercise training and weight loss impacts glucose tolerance in older men. J. Clin. Endocrinol. Metab..

[79] Dubé M.-C., Lemieux S., Piché M.-E., Corneau L., Bergeron J., Riou M.-E., Weisnagel S.J. (2011). The contribution of visceral adiposity and mid-thigh fat-rich muscle to the metabolic profile in postmenopausal women. Obesity (Silver Spring).

[80] Durheim M.T., Slentz C.A., Bateman L.A., Mabe S.K., Kraus W.E. (2008). Relationships between exercise-induced reductions in thigh intermuscular adipose tissue, changes in lipoprotein particle size, and visceral adiposity. Am. J. Physiol. Endocrinol. Metab..

[81] Goodpaster B.H., Carlson C.L., Visser M., Kelley D.E., Scherzinger A., Harris T.B., Stamm E., Newman A.B. (2001). Attenuation of skeletal muscle and strength in the elderly: The Health ABC Study. J. Appl. Physiol..

[82] Yoshida Y., Marcus R.L., Lastayo P.C. (2012). Intramuscular adipose tissue and central activation in older adults. Muscle Nerve.

[83] Hughes V.A., Roubenoff R., Wood M., Frontera W.R., Evans W.J., Fiatarone Singh M.A. (2004). Anthropometric assessment of 10-y changes in body composition in the elderly. Am. J. Clin. Nutr..

[84] Raguso C.A., Kyle U., Kossovsky M.P., Roynette C., Paoloni-Giacobino A., Hans D., Genton L., Pichard C. (2006). A 3-year longitudinal study on body composition changes in the elderly: Role of physical exercise. Clin. Nutr..

[85] Ng A.C., Melton L.J., Atkinson E.J., Achenbach S.J., Holets M.F., Peterson J.M., Khosla S., Drake M.T. (2013). Relationship of adiposity to bone volumetric density and microstructure in men and women across the adult lifespan. Bone.

[86] Haffner S.M., Katz M.S., Stern M.P., Dunn J.F. (1989). Relationship of sex hormone binding globulin to overall adiposity and body fat distribution in a biethnic population. Int. J. Obes..

[87] MacDonald P.C., Edman C.D., Hemsell D.L., Porter J.C., Siiteri P.K. (1978). Effect of obesity on conversion of plasma androstenedione to estrone in postmenopausal women with and without endometrial cancer. Am. J. Obstet. Gynecol..

[88] Cleland W.H., Mendelson C.R., Simpson E.R. (1985). Effects of aging and obesity on aromatase activity of human adipose cells. J. Clin. Endocrinol. Metab..

[89] Napoli N., Vattikuti S., Yarramaneni J., Giri T.K., Nekkalapu S., Qualls C., Armamento-Villareal R.C. (2012). Increased 2-hydroxylation of estrogen is associated with lower body fat and increased lean body mass in postmenopausal women. Maturitas.

[90] Hannemann A., Breer S., Wallaschofski H., Nauck M., Baumeister S.E., Barvencik F., Amling M., Schinke T., Haring R., Keller J. (2013). Osteocalcin is associated with testosterone in the general population and selected patients with bone disorders. Andrology.

[91] Ferron M., Wei J., Yoshizawa T., Del Fattore A., DePinho R.A., Teti A., Ducy P., Karsenty G. (2010). Insulin signaling in osteoblasts integrates bone remodeling and energy metabolism. Cell.

[92] Oury F., Sumara G., Sumara O., Ferron M., Chang H., Smith C.E., Hermo L., Suarez S., Roth B.L. (2011). Endocrine regulation of male fertility by the skeleton. Cell.

[93] Cawthon P.M., Shahnazari M., Orwoll E.S., Lane N.E. (2016). Osteoporosis in men: Findings from the Osteoporotic Fractures in Men Study (MrOS). Ther. Adv. Musculoskelet. Dis..

[94] Seeman E. (2002). Pathogenesis of bone fragility in women and men. Lancet.

[95] Sundh D., Mellström D., Nilsson M., Karlsson M., Ohlsson C., Lorentzon M. (2015). Increased cortical porosity in older men with fracture. J. Bone Miner. Res..

[96] Donaldson L.J., Cook A., Thomson R.G. (1990). Incidence of fractures in a geographically defined population. J. Epidemiol. Community Health.

[97] Karsenty G. (2012). The mutual dependence between bone and gonads. J. Endocrinol..

[98] Khosla S., Melton L.J., Atkinson E.J., O’Fallon W.M. (2001). Relationship of serum sex steroid levels to longitudinal changes in bone density in young *vs.* elderly men. J. Clin. Endocrinol. Metab..

[99] Riggs B.L., Khosla S., Melton L.J. (2002). Sex steroids and the construction and conservation of the adult skeleton. Endocr. Rev..

[100] Nakamura T., Imai Y., Matsumoto T., Sato S., Takeuchi K., Igarashi K., Harada Y., Azuma Y., Krust A., Yamamoto Y. (2007). Estrogen prevents bone loss via estrogen receptor alpha and induction of Fas ligand in osteoclasts. Cell.

[101] Bobjer J., Bogefors K., Isaksson S., Leijonhufvud I., Åkesson K., Giwercman Y.L., Giwercman A. (2016). High prevalence of hypogonadism and associated impaired metabolic and bone mineral status in subfertile men. Clin. Endocrinol..

[102] Haring R., Völzke H., Felix S.B., Schipf S., Dörr M., Rosskopf D., Nauck M., Schöfl C., Wallaschofski H. (2009). Prediction of metabolic syndrome by low serum testosterone levels in men: results from the study of health in Pomerania. Diabetes.

[103] Yassin A., Nettleship J.E., Talib R.A., Almehmadi Y., Doros G. (2016). Effects of testosterone replacement therapy withdrawal and re-treatment in hypogonadal elderly men upon obesity, voiding function and prostate safety parameters. Aging Male.

[104] Nieschlag E. (2015). Current topics in testosterone replacement of hypogonadal men. Best Pract. Res. Clin. Endocrinol. Metab..

[105] Prats-Puig A., Mas-Parareda M., Riera-Pérez E., González-Forcadell D., Mier C., Mallol-Guisset M., Díaz M., Bassols J., de Zegher F., Ibáñez L. (2010). Carboxylation of osteocalcin affects its association with metabolic parameters in healthy children. Diabetes Care.

[106] Franceschi C., Capri M., Monti D., Giunta S., Olivieri F., Sevini F., Panourgia M.P., Invidia L., Celani L., Scurti M. (2007). Inflammaging and anti-inflammaging: A systemic perspective on aging and longevity emerged from studies in humans. Mech. Ageing Dev..

[107] Targownik L.E., Bernstein C.N., Leslie W.D. (2013). Inflammatory bowel disease and the risk of osteoporosis and fracture. Maturitas.

[108] Gautier A., Bonnet F., Dubois S., Massart C., Grosheny C., Bachelot A., Aubé C., Balkau B., Ducluzeau P.-H. (2013). Associations between visceral adipose tissue, inflammation and sex steroid concentrations in men. Clin. Endocrinol..

[109] Weyer C., Funahashi T., Tanaka S., Hotta K., Matsuzawa Y., Pratley R.E., Tataranni P.A. (2001). Hypoadiponectinemia in obesity and type 2 diabetes: Close association with insulin resistance and hyperinsulinemia. J. Clin. Endocrinol. Metab..

[110] Lenchik L., Register T.C., Hsu F.C., Lohman K., Nicklas B.J., Freedman B.I., Langefeld C.D., Carr J.J., Bowden D.W. (2003). Adiponectin as a novel determinant of bone mineral density and visceral fat. Bone.

[111] Yamauchi T., Kamon J., Waki H., Terauchi Y., Kubota N., Hara K., Mori Y., Ide T., Murakami K., Tsuboyama-Kasaoka N. (2001). The fat-derived hormone adiponectin reverses insulin resistance associated with both lipoatrophy and obesity. Nat. Med..

[112] Berner H.S., Lyngstadaas S.P., Spahr A., Monjo M., Thommesen L., Drevon C.A., Syversen U., Reseland J.E. (2004). Adiponectin and its receptors are expressed in bone-forming cells. Bone.

[113] Wang Y., Lam K.S.L., Xu J.Y., Lu G., Xu L.Y., Cooper G.J.S., Xu A. (2005). Adiponectin inhibits cell proliferation by interacting with several growth factors in an oligomerization-dependent manner. J. Biol. Chem..

[114] Williams G.A., Wang Y., Callon K.E., Watson M., Lin J., Lam J.B.B., Costa J.L., Orpe A., Broom N., Naot D. (2009). *In vitro* and *in vivo* effects of adiponectin on bone. Endocrinology.

[115] Richards J.B., Valdes A.M., Burling K., Perks U.C., Spector T.D. (2007). Serum adiponectin and bone mineral density in women. J. Clin. Endocrinol. Metab..

[116] Jürimäe J., Jürimäe T. (2007). Plasma adiponectin concentration in healthy pre- and postmenopausal women: Relationship with body composition, bone mineral, and metabolic variables. Am. J. Physiol. Endocrinol. Metab..

[117] Peng X.-D., Xie H., Zhao Q., Wu X.-P., Sun Z.-Q., Liao E.-Y. (2008). Relationships between serum adiponectin, leptin, resistin, visfatin levels and bone mineral density, and bone biochemical markers in Chinese men. Clin. Chim. Acta.

[118] Napoli N., Pedone C., Pozzilli P., Lauretani F., Ferrucci L., Incalzi R.A. (2010). Adiponectin and bone mass density: The InCHIANTI study. Bone.

[119] Tamura T., Yoneda M., Yamane K., Nakanishi S., Nakashima R., Okubo M., Kohno N. (2007). Serum leptin and adiponectin are positively associated with bone mineral density at the distal radius in patients with type 2 diabetes mellitus. Metabolism.

[120] Kontogianni M.D., Dafni U.G., Routsias J.G., Skopouli F.N. (2004). Blood leptin and adiponectin as possible mediators of the relation between fat mass and BMD in perimenopausal women. J. Bone Miner. Res..

[121] Pasco J.A., Henry M.J., Kotowicz M.A., Collier G.R., Ball M.J., Ugoni A.M., Nicholson G.C. (2001). Serum leptin levels are associated with bone mass in nonobese women. J. Clin. Endocrinol. Metab..

[122] Elefteriou F., Takeda S., Ebihara K., Magre J., Patano N., Kim C.A., Ogawa Y., Liu X., Ware S.M., Craigen W.J. (2004). Serum leptin level is a regulator of bone mass. Proc. Natl. Acad. Sci. USA.

[123] Ducy P., Amling M., Takeda S., Priemel M., Schilling A.F., Beil F.T., Shen J., Vinson C., Rueger J.M., Karsenty G. (2000). Leptin inhibits bone formation through a hypothalamic relay: A central control of bone mass. Cell.

[124] Blain H., Vuillemin A., Guillemin F., Durant R., Hanesse B., de Talance N., Doucet B., Jeandel C. (2002). Serum leptin level is a predictor of bone mineral density in postmenopausal women. J. Clin. Endocrinol. Metab..

[125] Shaarawy M., Abassi A.F., Hassan H., Salem M.E. (2003). Relationship between serum leptin concentrations and bone mineral density as well as biochemical markers of bone turnover in women with postmenopausal osteoporosis. Fertil. Steril..

[126] Holecki M., Wiecek A. (2010). Relationship between body fat mass and bone metabolism. Pol. Arch. Med. Wewn..

[127] Couce M.E., Green D., Brunetto A., Achim C., Lloyd R.V., Burguera B. (2001). Limited brain access for leptin in obesity. Pituitary.

[128] Lamghari M., Tavares L., Camboa N., Barbosa M.A. (2006). Leptin effect on RANKL and OPG expression in MC3T3-E1 osteoblasts. J. Cell. Biochem..

[129] Shintani M., Ogawa Y., Ebihara K., Aizawa-Abe M., Miyanaga F., Takaya K., Hayashi T., Inoue G., Hosoda K., Kojima M. (2001). Ghrelin, an endogenous growth hormone secretagogue, is a novel orexigenic peptide that antagonizes leptin action through the activation of hypothalamic neuropeptide Y/Y1 receptor pathway. Diabetes.

[130] Wren A.M., Small C.J., Abbott C.R., Dhillo W.S., Seal L.J., Cohen M.A., Batterham R.L., Taheri S., Stanley S.A., Ghatei M.A. (2001). Ghrelin causes hyperphagia and obesity in rats. Diabetes.

[131] Wren A.M., Seal L.J., Cohen M.A., Brynes A.E., Frost G.S., Murphy K.G., Dhillo W.S., Ghatei M.A., Bloom S.R. (2001). Ghrelin enhances appetite and increases food intake in humans. J. Clin. Endocrinol. Metab..

[132] Asakawa A., Inui A., Kaga T., Yuzuriha H., Nagata T., Ueno N., Makino S., Fujimiya M., Niijima A., Fujino M.A. (2001). Ghrelin is an appetite-stimulatory signal from stomach with structural resemblance to motilin. Gastroenterology.

[133] Tschöp M., Smiley D.L., Heiman M.L. (2000). Ghrelin induces adiposity in rodents. Nature.

[134] Fukushima N., Hanada R., Teranishi H., Fukue Y., Tachibana T., Ishikawa H., Takeda S., Takeuchi Y., Fukumoto S., Kangawa K. (2005). Ghrelin directly regulates bone formation. J. Bone Miner. Res..

[135] Tavassoli M. (1984). Marrow adipose cells and hemopoiesis: An interpretative review. Exp. Hematol..

[136] Rosen C.J., Bouxsein M.L. (2006). Mechanisms of disease: Is osteoporosis the obesity of bone?. Nat. Clin. Pract. Rheumatol..

[137] Rosen C.J., Klibanski A. (2009). Bone, fat, and body composition: Evolving concepts in the pathogenesis of osteoporosis. Am. J. Med..

[138] Takeda S., Elefteriou F., Karsenty G. (2003). Common endocrine control of body weight, reproduction, and bone mass. Annu. Rev. Nutr..

[139] Schellinger D., Lin C.S., Hatipoglu H.G., Fertikh D. (2001). Potential value of vertebral proton MR spectroscopy in determining bone weakness. Am. J. Neuroradiol..

[140] Bredella M.A., Gill C.M., Gerweck A.V., Landa M.G., Kumar V., Daley S.M., Torriani M., Miller K.K. (2013). Ectopic and serum lipid levels are positively associated with bone marrow fat in obesity. Radiology.

[141] Parhami F. (2003). Possible role of oxidized lipids in osteoporosis: Could hyperlipidemia be a risk factor?. Prostaglandins Leukot. Essent. Fat. Acids.

[142] Rajamannan N.M. (2008). Low-density lipoprotein and aortic stenosis. Heart.

[143] Zernicke R.F., Salem G.J., Barnard R.J., Schramm E. (1995). Long-term, high-fat-sucrose diet al.ters rat femoral neck and vertebral morphology, bone mineral content, and mechanical properties. Bone.

[144] Demigné C., Bloch-Faure M., Picard N., Sabboh H., Besson C., Rémésy C., Geoffroy V., Gaston A.-T., Nicoletti A., Hagège A. (2006). Mice chronically fed a westernized experimental diet as a model of obesity, metabolic syndrome and osteoporosis. Eur. J. Nutr..

[145] Woo D.G., Lee B.Y., Lim D., Kim H.S. (2009). Relationship between nutrition factors and osteopenia: Effects of experimental diets on immature bone quality. J. Biomech..

[146] Patsch J.M., Kiefer F.W., Varga P., Pail P., Rauner M., Stupphann D., Resch H., Moser D., Zysset P.K., Stulnig T.M. (2011). Increased bone resorption and impaired bone microarchitecture in short-term and extended high-fat diet-induced obesity. Metabolism.

[147] Cao J.J., Sun L., Gao H. (2010). Diet-induced obesity alters bone remodeling leading to decreased femoral trabecular bone mass in mice. Ann. N. Y. Acad. Sci..

[148] Ionova-Martin S.S., Wade J.M., Tang S., Shahnazari M., Ager J.W., Lane N.E., Yao W., Alliston T., Vaisse C., Ritchie R.O. (2011). Changes in cortical bone response to high-fat diet from adolescence to adulthood in mice. Osteoporos. Int..

[149] Lorincz C., Reimer R.A., Boyd S.K., Zernicke R.F. (2010). High-fat, sucrose diet impairs geometrical and mechanical properties of cortical bone in mice. Br. J. Nutr..

[150] Zernicke R.F., Salem G.J., Barnard R.J., Woodward J.S., Meduski J.W., Meduski J.D. (1995). Adaptations of immature trabecular bone to exercise and augmented dietary protein. Med. Sci. Sports Exerc..

[151] Wu W.X., Glasier A., Norman J., Kelly R.W., Baird D.T., McNeilly A.S. (1990). The effects of the antiprogestin mifepristone, *in vivo*, and progesterone *in vitro* on prolactin production by the human decidua in early pregnancy. Hum. Reprod..

[152] Tsanzi E., Light H.R., Tou J.C. (2008). The effect of feeding different sugar-sweetened beverages to growing female Sprague-Dawley rats on bone mass and strength. Bone.

[153] Yarrow J.F., Toklu H.Z., Balaez A., Phillips E.G., Otzel D.M., Chen C., Wronski T.J., Aguirre J.I., Sakarya Y., Tümer N. (2016). Fructose consumption does not worsen bone deficits resulting from high-fat feeding in young male rats. Bone.

[154] Fried A., Manske S.L., Eller L.K., Lorincz C., Reimer R.A., Zernicke R.F. (2012). Skim milk powder enhances trabecular bone architecture compared with casein or whey in diet-induced obese rats. Nutrition.

[155] Farina E.K., Kiel D.P., Roubenoff R., Schaefer E.J., Cupples L.A., Tucker K.L. (2011). Protective effects of fish intake and interactive effects of long-chain polyunsaturated fatty acid intakes on hip bone mineral density in older adults: The Framingham Osteoporosis Study. Am. J. Clin. Nutr..

[156] Farina E.K., Kiel D.P., Roubenoff R., Schaefer E.J., Cupples L.A., Tucker K.L. (2012). Plasma phosphatidylcholine concentrations of polyunsaturated fatty acids are differentially associated with hip bone mineral density and hip fracture in older adults: The Framingham Osteoporosis Study. J. Bone Miner. Res..

[157] Martin-Bautista E., Muñoz-Torres M., Fonolla J., Quesada M., Poyatos A., Lopez-Huertas E. (2010). Improvement of bone formation biomarkers after 1-year consumption with milk fortified with eicosapentaenoic acid, docosahexaenoic acid, oleic acid, and selected vitamins. Nutr. Res..

[158] Lappe J., Kunz I., Bendik I., Prudence K., Weber P., Recker R., Heaney R.P. (2013). Effect of a combination of genistein, polyunsaturated fatty acids and vitamins D3 and K1 on bone mineral density in postmenopausal women: A randomized, placebo-controlled, double-blind pilot study. Eur. J. Nutr..

[159] Mangano K.M., Sahni S., Kerstetter J.E., Kenny A.M., Hannan M.T. (2013). Polyunsaturated fatty acids and their relation with bone and muscle health in adults. Curr. Osteoporos. Rep..

[160] Weaver C.M., Gordon C.M., Janz K.F., Kalkwarf H.J., Lappe J.M., Lewis R., O’Karma M., Wallace T.C., Zemel B.S. (2016). The National Osteoporosis Foundation’s position statement on peak bone mass development and lifestyle factors: A systematic review and implementation recommendations. Osteoporos. Int..

[161] Alexy U., Remer T., Manz F., Neu C.M., Schoenau E. (2005). Long-term protein intake and dietary potential renal acid load are associated with bone modeling and remodeling at the proximal radius in healthy children. Am. J. Clin. Nutr..

[162] Remer T., Manz F., Alexy U., Schoenau E., Wudy S.A., Shi L. (2011). Long-term high urinary potential renal acid load and low nitrogen excretion predict reduced diaphyseal bone mass and bone size in children. J. Clin. Endocrinol. Metab..

[163] Bounds W., Skinner J., Carruth B.R., Ziegler P. (2005). The relationship of dietary and lifestyle factors to bone mineral indexes in children. J. Am. Diet. Assoc..

[164] Vatanparast H., Bailey D.A., Baxter-Jones A.D.G., Whiting S.J. (2007). The effects of dietary protein on bone mineral mass in young adults may be modulated by adolescent calcium intake. J. Nutr..

[165] Hoppe C., Mølgaard C., Michaelsen K.F. (2000). Bone size and bone mass in 10-year-old Danish children: Effect of current diet. Osteoporos. Int..

[166] Iuliano-Burns S., Stone J., Hopper J.L., Seeman E. (2005). Diet and exercise during growth have site-specific skeletal effects: A co-twin control study. Osteoporos. Int..

[167] Chevalley T., Bonjour J.-P., Ferrari S., Rizzoli R. (2008). High-protein intake enhances the positive impact of physical activity on BMC in prepubertal boys. J. Bone Miner. Res..

[168] Marotte C., Bryk G., Gonzales Chaves M.M.S., Lifshitz F., de Portela M.L.P.M., Zeni S.N. (2014). Low dietary calcium and obesity: A comparative study in genetically obese and normal rats during early growth. Eur. J. Nutr..

[169] Reid I.R. (1996). Therapy of osteoporosis: Calcium, vitamin D, and exercise. Am. J. Med. Sci..

[170] Salamone L.M., Cauley J.A., Black D.M., Simkin-Silverman L., Lang W., Gregg E., Palermo L., Epstein R.S., Kuller L.H., Wing R. (1999). Effect of a lifestyle intervention on bone mineral density in premenopausal women: A randomized trial. Am. J. Clin. Nutr..

[171] Langlois J.A., Mussolino M.E., Visser M., Looker A.C., Harris T., Madans J. (2001). Weight loss from maximum body weight among middle-aged and older white women and the risk of hip fracture: The NHANES I epidemiologic follow-up study. Osteoporos. Int..

[172] Ensrud K.E., Fullman R.L., Barrett-Connor E., Cauley J.A., Stefanick M.L., Fink H.A., Lewis C.E., Orwoll E. (2005). Voluntary weight reduction in older men increases hip bone loss: The osteoporotic fractures in men study. J. Clin. Endocrinol. Metab..

[173] Bleicher K., Cumming R.G., Naganathan V., Travison T.G., Sambrook P.N., Blyth F.M., Handelsman D.J., Le Couteur D.G., Waite L.M., Creasey H.M. (2011). The role of fat and lean mass in bone loss in older men: Findings from the CHAMP study. Bone.

[174] Hawkins J., Cifuentes M., Pleshko N.L., Ambia-Sobhan H., Shapses S.A. (2010). Energy restriction is associated with lower bone mineral density of the tibia and femur in lean but not obese female rats. J. Nutr..

[175] Talbott S.M., Cifuentes M., Dunn M.G., Shapses S.A. (2001). Energy restriction reduces bone density and biomechanical properties in aged female rats. J. Nutr..

[176] Devlin M.J., Stetter C.M., Lin H.-M., Beck T.J., Legro R.S., Petit M.A., Lieberman D.E., Lloyd T. (2010). Peripubertal estrogen levels and physical activity affect femur geometry in young adult women. Osteoporos. Int..

[177] Labouesse M.A., Gertz E.R., Piccolo B.D., Souza E.C., Schuster G.U., Witbracht M.G., Woodhouse L.R., Adams S.H., Keim N.L., Van Loan M.D. (2014). Associations among endocrine, inflammatory, and bone markers, body composition and weight loss induced bone loss. Bone.

[178] Zibellini J., Seimon R.V., Lee C.M., Gibson A.A., Hsu M.S., Shapses S.A., Nguyen T.V., Sainsbury A. (2015). Does diet-induced weight loss lead to bone loss in overweight or obese adults? A systematic review and meta-analysis of clinical trials. J. Bone Miner. Res..

[179] Pop L.C., Sukumar D., Tomaino K., Schlussel Y., Schneider S.H., Gordon C.L., Wang X., Shapses S.A. (2015). Moderate weight loss in obese and overweight men preserves bone quality. Am. J. Clin. Nutr..

[180] Skerry T.M. (2008). The response of bone to mechanical loading and disuse: Fundamental principles and influences on osteoblast/osteocyte homeostasis. Arch. Biochem. Biophys..

[181] Villareal D.T., Chode S., Parimi N., Sinacore D.R., Hilton T., Armamento-Villareal R., Napoli N., Qualls C., Shah K. (2011). Weight loss, exercise, or both and physical function in obese older adults. N. Engl. J. Med..

[182] Shah K., Armamento-Villareal R., Parimi N., Chode S., Sinacore D.R., Hilton T.N., Napoli N., Qualls C., Villareal D.T. (2011). Exercise training in obese older adults prevents increase in bone turnover and attenuates decrease in hip bone mineral density induced by weight loss despite decline in bone-active hormones. J. Bone Miner. Res..

[183] Daly R.M., Dunstan D.W., Owen N., Jolley D., Shaw J.E., Zimmet P.Z. (2005). Does high-intensity resistance training maintain bone mass during moderate weight loss in older overweight adults with type 2 diabetes?. Osteoporos. Int..

[184] Reid I.R., Legge M., Stapleton J.P., Evans M.C., Grey A.B. (1995). Regular exercise dissociates fat mass and bone density in premenopausal women. J. Clin. Endocrinol. Metab..

[185] Meyer H.E., Willett W.C., Flint A.J., Feskanich D. (2016). Abdominal obesity and hip fracture: Results from the Nurses’ Health Study and the Health Professionals Follow-Up Study. Osteoporos. Int..

[186] Endocrine Disruption. https://www.epa.gov/endocrine-disruption.

[187] Birnbaum L.S. (2013). When environmental chemicals act like uncontrolled medicine. Trends Endocrinol. Metab..

[188] Thayer K.A., Heindel J.J., Bucher J.R., Gallo M.A. (2012). Role of environmental chemicals in diabetes and obesity: a National Toxicology Program workshop review. Environ. Health Perspect..

[189] Kopras E., Potluri V., Bermudez M.-L., Williams K., Belcher S., Kasper S. (2014). Actions of endocrine-disrupting chemicals on stem/progenitor cells during development and disease. Endocr. Relat. Cancer.

[190] Tsukamoto Y., Ishihara Y., Miyagawa-Tomita S., Hagiwara H. (2004). Inhibition of ossification *in vivo* and differentiation of osteoblasts *in vitro* by tributyltin. Biochem. Pharmacol..

[191] Salmela E., Sahlberg C., Alaluusua S., Lukinmaa P.-L. (2008). Tributyltin impairs dentin mineralization and enamel formation in cultured mouse embryonic molar teeth. Toxicol. Sci..

[192] Salmela E., Alaluusua S., Sahlberg C., Lukinmaa P.-L. (2012). Tributyltin alters osteocalcin, matrix metalloproteinase 20 and dentin sialophosphoprotein gene expression in mineralizing mouse embryonic tooth *in vitro*. Cells Tissues Organs.

[193] Grün F., Blumberg B. (2006). (2006) Environmental obesogens: organotins and endocrine disruption via nuclear receptor signaling. Endocrinology.

[194] Grün F., Watanabe H., Zamanian Z., Maeda L., Arima K., Cubacha R., Gardiner D.M., Kanno J., Iguchi T., Blumberg B. (2006). Endocrine-disrupting organotin compounds are potent inducers of adipogenesis in vertebrates. Mol. Endocrinol..

[195] Choi W.-J., Han S.-H. (2015). Blood cadmium is associated with osteoporosis in obese males but not in non-obese males: The Korea National Health and Nutrition Examination Survey 2008–2011. Int. J. Environ. Res. Public Health.

[196] Gupta V. (2012). Pleiotropic effects of incretins. Indian J. Endocrinol. Metab..

[197] Faienza M.F., Luce V., Ventura A., Colaianni G., Colucci S., Cavallo L., Grano M., Brunetti G. (2015). Skeleton and glucose metabolism: A bone-pancreas loop. Int. J. Endocrinol..

[198] Baggio L.L., Drucker D.J. (2007). Biology of incretins: GLP-1 and GIP. Gastroenterology.

[199] Zhong Q., Itokawa T., Sridhar S., Ding K.-H., Xie D., Kang B., Bollag W.B., Bollag R.J., Hamrick M., Insogna K. (2007). Effects of glucose-dependent insulinotropic peptide on osteoclast function. Am. J. Physiol. Endocrinol. Metab..

[200] Pacheco-Pantoja E.L., Ranganath L.R., Gallagher J.A., Wilson P.J.M., Fraser W.D. (2011). Receptors and effects of gut hormones in three osteoblastic cell lines. BMC Physiol..

[201] Nuche-Berenguer B., Portal-Núñez S., Moreno P., González N., Acitores A., López-Herradón A., Esbrit P., Valverde I., Villanueva-Peñacarrillo M.L. (2010). Presence of a functional receptor for GLP-1 in osteoblastic cells, independent of the cAMP-linked GLP-1 receptor. J. Cell. Physiol..

[202] Yamada Y. (2009). Incretin and bone. Clin. Calcium.

[203] Nuche-Berenguer B., Moreno P., Esbrit P., Dapía S., Caeiro J.R., Cancelas J., Haro-Mora J.J., Villanueva-Peñacarrillo M.L. (2009). Effect of GLP-1 treatment on bone turnover in normal, type 2 diabetic, and insulin-resistant states. Calcif. Tissue Int..

[204] Nuche-Berenguer B., Moreno P., Portal-Nuñez S., Dapía S., Esbrit P., Villanueva-Peñacarrillo M.L. (2010). Exendin-4 exerts osteogenic actions in insulin-resistant and type 2 diabetic states. Regul. Pept..

[205] Nuche-Berenguer B., Lozano D., Gutiérrez-Rojas I., Moreno P., Mariñoso M.L., Esbrit P., Villanueva-Peñacarrillo M.L. (2011). GLP-1 and exendin-4 can reverse hyperlipidic-related osteopenia. J. Endocrinol..

[206] Pereira M., Jeyabalan J., Jørgensen C.S., Hopkinson M., Al-Jazzar A., Roux J.P., Chavassieux P., Orriss I.R., Cleasby M.E., Chenu C. (2015). Chronic administration of Glucagon-like peptide-1 receptor agonists improves trabecular bone mass and architecture in ovariectomised mice. Bone.

[207] Lu N., Sun H., Yu J., Wang X., Liu D., Zhao L., Sun L., Zhao H., Tao B., Liu J. (2015). Glucagon-like peptide-1 receptor agonist Liraglutide has anabolic bone effects in ovariectomized rats without diabetes. PLoS ONE.

[208] Henriksen D.B., Alexandersen P., Hartmann B., Adrian C.L., Byrjalsen I., Bone H.G., Holst J.J. (2009). Christiansen C Four-month treatment with GLP-2 significantly increases hip BMD: A randomized, placebo-controlled, dose-ranging study in postmenopausal women with low BMD. Bone.

[209] Bunck M.C., Eliasson B., Cornér A., Heine R.J., Shaginian R.M., Taskinen M.-R., Yki-Järvinen H., Smith U., Diamant M. (2011). Exenatide treatment did not affect bone mineral density despite body weight reduction in patients with type 2 diabetes. Diabetes Obes. Metab..

[210] Gilbert M.P., Marre M., Holst J.J., Garber A., Baeres F.M.M., Thomsen H., Pratley R.E. (2015). Comparison of the long-term effects of liraglutide and glimepiride monotherapy on bone mineral density in patients with type 2 diabetes. Endocr. Pract..

[211] Iepsen E.W., Lundgren J.R., Hartmann B., Pedersen O., Hansen T., Jørgensen N.R., Jensen J.-E.B., Holst J.J., Madsbad S., Torekov S.S. (2015). GLP-1 Receptor agonist treatment increases bone formation and prevents bone loss in weight-reduced obese women. J. Clin. Endocrinol. Metab..

[212] Mabilleau G., Mieczkowska A., Chappard D. (2014). Use of glucagon-like peptide-1 receptor agonists and bone fractures: A meta-analysis of randomized clinical trials (-1:meta). J. Diabetes.

[213] Su B., Sheng H., Zhang M., Bu L., Yang P., Li L., Li F., Sheng C., Han Y., Qu S., Wang J. (2014). Risk of bone fractures associated with glucagon-like peptide-1 receptor agonists’ treatment: A meta-analysis of randomized controlled trials. Endocrine.

[214] Pace D.G., Blotner S., Guerciolini R. (2001). Short-term orlistat treatment does not affect mineral balance and bone turnover in obese men. J. Nutr..

[215] Gotfredsen A., Westergren Hendel H., Andersen T. (2001). Influence of orlistat on bone turnover and body composition. Int. J. Obes. Relat. Metab. Disord..

[216] Compston J.E., Laskey M.A., Croucher P.I., Coxon A., Kreitzman S. (1992). Effect of diet-induced weight loss on total body bone mass. Clin. Sci..

[217] Brzozowska M.M., Sainsbury A., Eisman J.A., Baldock P.A., Center J.R. (2013). Bariatric surgery, bone loss, obesity and possible mechanisms. Obes. Rev..

[218] Andersen R.E., Wadden T.A., Herzog R.J. (1997). Changes in bone mineral content in obese dieting women. Metabolism.

[219] Jensen L.B., Kollerup G., Quaade F., Sørensen O.H. (2001). Bone minerals changes in obese women during a moderate weight loss with and without calcium supplementation. J. Bone Miner. Res..

[220] Svendsen O.L., Hassager C., Christiansen C. (1993). Effect of an energy-restrictive diet, with or without exercise, on lean tissue mass, resting metabolic rate, cardiovascular risk factors, and bone in overweight postmenopausal women. Am. J. Med..

[221] Riedt C.S., Cifuentes M., Stahl T., Chowdhury H.A., Schlussel Y., Shapses S.A. (2005). Overweight postmenopausal women lose bone with moderate weight reduction and 1 g/day calcium intake. J. Bone Miner. Res..

[222] Ricci T.A., Chowdhury H.A., Heymsfield S.B., Stahl T., Pierson R.N., Shapses S.A. (1998). Calcium supplementation suppresses bone turnover during weight reduction in postmenopausal women. J. Bone Miner. Res..

[223] Ramsdale S.J., Bassey E.J. (1994). Changes in bone mineral density associated with dietary-induced loss of body mass in young women. Clin. Sci..

[224] Van Loan M.D., Johnson H.L., Barbieri T.F. (1998). Effect of weight loss on bone mineral content and bone mineral density in obese women. Am. J. Clin. Nutr..

[225] Shapses S.A., Von Thun N.L., Heymsfield S.B., Ricci T.A., Ospina M., Pierson R.N., Stahl T. (2001). Bone turnover and density in obese premenopausal women during moderate weight loss and calcium supplementation. J. Bone Miner. Res..

[226] Riedt C.S., Schlussel Y., von Thun N., Ambia-Sobhan H., Stahl T., Field M.P., Sherrell R.M., Shapses S.A. (2007). Premenopausal overweight women do not lose bone during moderate weight loss with adequate or higher calcium intake. Am. J. Clin. Nutr..

[227] Pritchard J.E., Nowson C.A., Wark J.D. (1996). Bone loss accompanying diet-induced or exercise-induced weight loss: A randomised controlled study. Int. J. Obes. Relat. Metab. Disord..

[228] Bakhireva L.N., Barrett-Connor E., Kritz-Silverstein D., Morton D.J. (2004). Modifiable predictors of bone loss in older men: A prospective study. Am. J. Prev. Med..

[229] Fogelholm G.M., Sievänen H.T., Kukkonen-Harjula T.K., Pasanen M.E. (2001). Bone mineral density during reduction, maintenance and regain of body weight in premenopausal, obese women. Osteoporos Int..

[230] Hamilton K.C., Fisher G., Roy J.L., Gower B.A., Hunter G.R. (2013). The effects of weight loss on relative bone mineral density in premenopausal women. Obesity.

[231] Villalon K.L., Gozansky W.S., van Pelt R.E., Wolfe P., Jankowski C.M., Schwartz R.S., Kohrt W.M. (2011). A losing battle: weight regain does not restore weight loss-induced bone loss in postmenopausal women. Obesity.

[232] Gower B.A., Casazza K. (2013). Divergent effects of obesity on bone health. J. Clin. Densitom..

[233] Armamento-Villareal R., Sadler C., Napoli N., Shah K., Chode S., Sinacore D.R., Qualls C., Villareal D.T. (2012). Weight loss in obese older adults increases serum sclerostin and impairs hip geometry but both are prevented by exercise training. J. Bone Miner. Res..

[234] Shapses S.A., Riedt C.S. (2006). Bone, body weight, and weight reduction: What are the concerns?. J. Nutr..

[235] Sundh D., Rudäng R., Zoulakis M., Nilsson A.G., Darelid A., Lorentzon M. (2016). A high amount of local adipose tissue is associated with high cortical porosity and low bone material strength in older women. J. Bone Miner. Res..

